# A New Model and Dating for the Evolution of Complex Plastids of Red Alga Origin

**DOI:** 10.1093/gbe/evae192

**Published:** 2024-09-06

**Authors:** Filip Pietluch, Paweł Mackiewicz, Kacper Ludwig, Przemysław Gagat

**Affiliations:** Department of Bioinformatics and Genomics, Faculty of Biotechnology, University of Wroclaw, 50-383 Wroclaw, Poland; Department of Bioinformatics and Genomics, Faculty of Biotechnology, University of Wroclaw, 50-383 Wroclaw, Poland; Department of Bioinformatics and Genomics, Faculty of Biotechnology, University of Wroclaw, 50-383 Wroclaw, Poland; Department of Bioinformatics and Genomics, Faculty of Biotechnology, University of Wroclaw, 50-383 Wroclaw, Poland

**Keywords:** Chromalveolata, Cryptophyta, endosymbiosis, phylogeny, plastid, stramenopiles

## Abstract

Complex plastids, characterized by more than two bounding membranes, still present an evolutionary puzzle for the traditional endosymbiotic theory. Unlike primary plastids that directly evolved from cyanobacteria, complex plastids originated from green or red algae. The Chromalveolata hypothesis proposes a single red alga endosymbiosis that involved the ancestor of all the Chromalveolata lineages: cryptophytes, haptophytes, stramenopiles, and alveolates. As extensive phylogenetic analyses contradict the monophyly of Chromalveolata, serial plastid endosymbiosis models were proposed, suggesting a single secondary red alga endosymbiosis within Cryptophyta, followed by subsequent plastid transfers to other chromalveolates. Our findings based on 97 plastid-encoded markers, 112 species, and robust phylogenetic methods challenge all the existing models. They reveal two independent secondary endosymbioses, one within Cryptophyta and one within stramenopiles, precisely the phylum Ochrophyta, with two different groups of red algae. Consequently, we propose a new model for the emergence of red alga plastid–containing lineages and, through molecular clock analyses, estimate their ages.

**Significance**

Cyanobacteria and eukaryote endosymbioses created a multitude of photosynthetic organelles called “plastids” that feed most life on our planet. For decades, scientists have been trying to untangle the puzzle of plastid evolution, i.e. when and how plastids were acquired and spread throughout the eukaryotic tree of life. Our new model, based on robust phylogenetic methods, challenges current hypotheses and offers fresh insights into the evolution of complex plastids of red alga origin in cryptophytes, haptophytes, stramenopiles, and alveolates.

## Introduction

The ability to convert solar energy into adenosine triphosphate is the distinctive feature of cyanobacteria and plastids. The first plastids were direct cyanobacteria descendants and are accordingly called “primary plastids.” They occur in glaucophytes, red algae, and green algae/land plants, which form the superassemblage Archaeplastida (Löffelhardt 2014; Burki et al. 2020). Primary plastids are surrounded by two membranes that correspond to those of their cyanobacterial ancestors. Interestingly, many protist groups, including euglenids, chlorarachniophytes, cryptophytes, haptophytes, stramenopiles, and alveolates, carry plastids enveloped by three or more membranes. They are called “complex plastids,” and their origin has been a topical issue in evolutionary biology for decades (Archibald 2015; Adl et al. 2019).

To explain the evolution of complex plastids, Cavalier-Smith (1999) proposed the Cabozoa and Chromalveolata hypotheses. The first suggested that plastids of euglenids and chlorarachniophytes were derived from a single green alga endosymbiosis, whereas the second postulated a single red alga endosymbiosis for the origin of plastids in cryptophytes, haptophytes, stramenopiles, and alveolates; the latter included dinoflagellates, perkinsids, colpodellids, and apicomplexans. The Cabozoa hypothesis was quickly refuted by phylogenetic analyses, which revealed that neither the host cells nor green alga endosymbionts of euglenids and chlorarachniophytes are closely related (Archibald and Keeling 2002; Rogers et al. 2007). In contrast, the Chromalveolata hypothesis influenced our understanding of endosymbiosis for over a decade (Keeling 2009; Gould et al. 2015). However, phylogenetic analyses also disproved this model as nuclear markers did not support the monophyly of the Chromalveolata clade (Burki et al. 2012, 2016; Strassert et al. 2021).

Subsequent models explaining the evolution of red alga–derived plastids and reconciling nuclear and plastid marker–based phylogenies involved serial endosymbioses. For example, Bodył et al. (2009a, 2009b, 2018) and Stiller et al. (2014) proposed that only the cryptophyte plastid directly descended from red algae, while the other red alga–derived plastids evolved through multiple transfers among the Chromalveolata lineages, with cryptophytes as their initial donors (supplementary fig. S1, Supplementary Material online).

As the evolution of Chromalveolata plastids remains unclear and the existing models are inconsistent, we decided to reinvestigate the history of red alga–derived plastids. Our phylogenies indicate that there are two independent red alga endosymbioses within chromalveolates, challenging the previous models. Consequently, we advance a new scenario for the evolution of red alga–derived plastids and employ molecular dating to estimate the age of the lineages containing them.

## Materials and Methods

We employed 97 conserved plastid-encoded proteins from 112 organisms, to include all major red alga plastid lineages, from the NCBI reference sequence database (O’Leary et al. 2016) and GenBank (Sayers et al. 2021; supplementary table S12, Supplementary Material online). Alignments were created using the L-INS-i algorithm in MAFFT (Katoh and Standley 2013) and assessed in AliView (Larsson 2014), and phylogenetically informative sites were selected with trimAl (Capella-Gutiérrez et al. 2009) and ClipKIT (Steenwyk et al. 2020) based on the benchmark provided by Steenwyk et al. (2020). To balance the phylogenetic signal and reduce noise from poorly aligned regions, we constructed phylogenetic trees using the ML method in IQ-TREE (Minh et al. 2020) and RAxML (Bouckaert et al. 2014), employing seven trimming strategies. Alignments were concatenated into supermatrices using SequenceMatrix (Vaidya et al. 2011) and compared with PhyKIT (Steenwyk et al. 2021; supplementary table S1, Supplementary Material online). An additional ML tree was inferred in IQ-TREE under the CAT model, and three phylogenies along with chronograms were calculated using the Bayesian approach, two in Beast (Bouckaert et al. 2014) and one in MrBayes (Ronquist et al. 2012). These analyses were based on the smart-gap trimming alignment, offering a supermatrix with the highest number of variables (26,449) and parsimony-informative sites (22,039) and preserving almost 97% of all parsimony-informative sites from the original alignment.

To address data heterogeneity, we considered 97 potential partitions within all supermatrices. Model selection was performed using ModelFinder (Kalyaanamoorthy et al. 2017) in IQ-TREE and PartitionFinder (Lanfear et al. 2017) for RAxML, MrBayes, and Beast (supplementary tables S13 to S29, Supplementary Material online). We also employed the CAT model in IQ-TREE (Lartillot and Philippe 2004). We tested variants with 10, 20, and 30 profiles using substitution matrices: Poisson, LG, and Q.yeast. Based on Bayesian Information Criterion, we selected the best-fit model: LG + C20 + F + R10. To further account for data heterogeneity, we employed only relaxed molecular clocks. In MrBayes, we used an independent gamma rate (IGR) model (Ronquist et al. 2012), while in Beast, we applied an uncorrelated lognormal model and an uncorrelated exponential model (Bouckaert et al. 2014). To test the assumptions of stationarity and homogeneity in our data, we performed the maximum test of symmetry in IQ-TREE for each partition. Using Benjamini–Hochberg correction for multiple testing, we found that all 17 partitions passed the test.

To assess the confidence of the inferred phylogenies, nonparametric bootstrap tests (100 replicates) were used for ML analyses in IQ-TREE (1,000 for the CAT model) and RAxML, while posterior probabilities were employed for Bayesian approaches. In Beast, the Yule tree prior and exponential priors were applied to define calibration constraints, while in MrBayes, offset exponential priors, in combination with the birth–death tree prior, were applied. During the Markov chain Monte Carlo process, tree samples were saved every 1,000 iterations. To ensure robust parameter estimation, the first 10% of samples were discarded as burnin. The median heights were calculated for the final chronograms.

## Results

### Phylogenetic Analyses

**Fig. 1.**
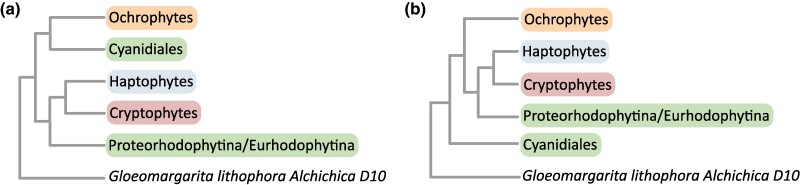
Two tree topologies obtained based on various trimming strategies (supplementary table S1, Supplementary Material online). In both trees, cryptophyte and haptophyte plastids are clustered with those from Proteorhodophytina/Eurhodophytina red algae, whereas stramenopile plastids are grouped either with Cyanidiales red algae a) or branch between Cyanidiales and the clade containing Proteorhodophytina/Eurhodophytina alongside Cryptophyta/Haptophyta b).

**Fig. 2.**
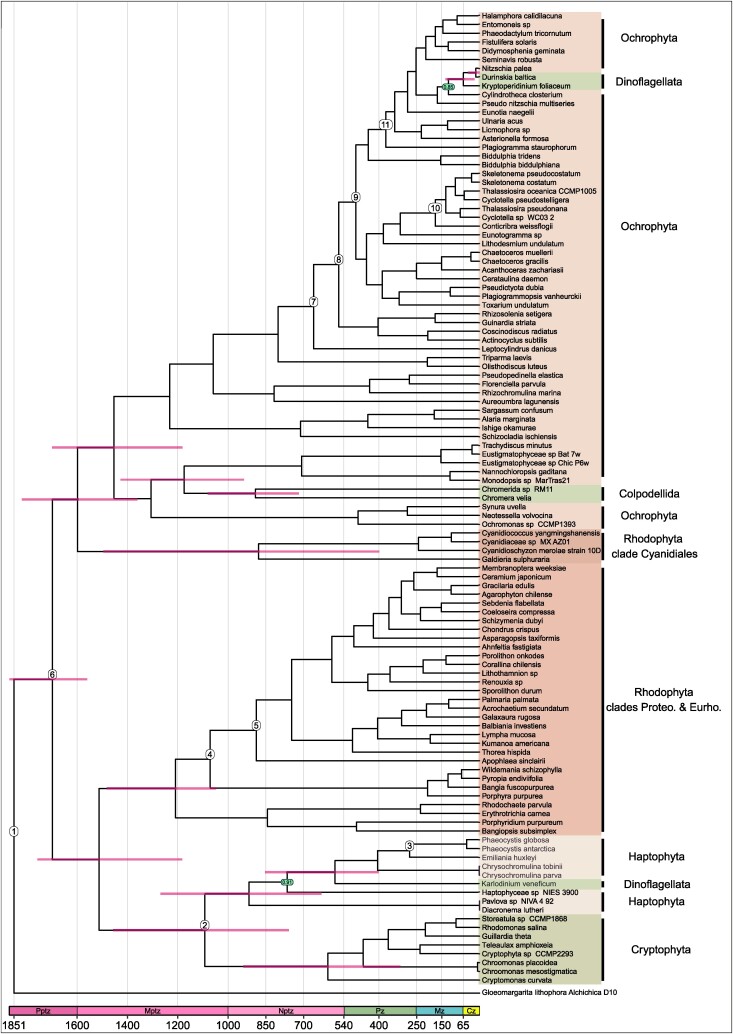
Time-calibrated phylogeny of red alga and red alga–derived plastids. The tree was inferred with Beast using an exponential model, based on ClipKIT smart-gap alignment (supplementary table S1, Supplementary Material online), along with substitution models provided in supplementary table S2, Supplementary Material online, and calibrated with constraints (numbers in white circles) listed in supplementary table S3, Supplementary Material online. Node numbers to help reading time estimates are given in supplementary fig. S21, Supplementary Material online, and the estimates in Table 2 and supplementary table S4, Supplementary Material online, and the bar rate at key nodes is 95% HPD (Highest Posterior Density). Nodes with posterior probability lower than 1 are marked with green circles. Abbreviations on the geological timescale for Eras: Cz—Cenozoic, Mz—Mesozoic, Pz—Paleozoic, Nptz—Neoproterozoic, Mptz—Mesoproterozoic, and Pptz—Paleoproterozoic.

All our phylogenies consistently rejected the monophyly of Chromalveolata plastids and showed that the clade of ochrophytes (photosynthetic stramenopiles) and cryptophytes + haptophytes (a mean bootstrap rate of 100% and posterior probability: 1) was polyphyletic. The trees provided strong to moderate support (bootstrap range: 61% to 88% and mean 78% and posterior probability range: 0.85 to 1 and mean 0.95) for the clustering of Cryptophyta and Haptophyta plastids with two red alga groups: Proteorhodophytina and Eurhodophytina (Figs. 1 and 2, supplementary figs. S2 to S20, Supplementary Material online). Ochrophyte plastids were either placed with thermoacidophilic red algae Cyanidiales (14 out of 20 trees) or branched between Cyanidiales and Proteorhodophytina/Eurhodophytina alongside with Cryptophyta/Haptophyta (6 out of 20 trees) (Figs. 1 and 2, supplementary figs. S2 to S20, Supplementary Material online, Table 1). The placement of ochrophytes with Cyanidiales (Fig. 1, topology A) achieved a maximum bootstrap rate of 68% in the IQ-TREE phylogeny based on the CAT model (supplementary fig. S20, Supplementary Material online), and two Bayesian trees provided a posterior probability of 1 for this clade (Fig. 2, supplementary fig. S3, Supplementary Material online). The alternative positioning of Ochrophyta (Fig. 1, topology B) reached a maximum bootstrap rate of 71% in the RAxML tree (supplementary fig. S18, Supplementary Material online). Considering the prevalence of the former topology in a larger number of trees, including the Bayesian ones, it appears to be more plausible.

**Table 1:** Results of phylogenetic analyses categorized by software, trimming strategy, and obtained topologies

Software	Trimming strategy^a^	Topology A	Topology B	Figure
Beast exponential	ClipKIT smart-gap	X		2
Beast lognormal	ClipKIT smart-gap	X		S2
MrBayes IGR	ClipKIT smart-gap	X		S3
IQ-TREE	ClipKIT gappy	X		S4
IQ-TREE	ClipKIT kpic	X		S5
IQ-TREE	ClipKIT kpic-smart-gap		X	S6
IQ-TREE	ClipKIT smart-gap	X		S7
IQ-TREE^b^	ClipKIT smart-gap	X		S20
IQ-TREE	trimAl automated1		X	S8
IQ-TREE	trimAl gappyout	X		S9
IQ-TREE	trimAl strict		X	S10
IQ-TREE	original alignment		X	S11
RAxML	ClipKIT gappy	X		S12
RAxML	ClipKIT kpic	X		S13
RAxML	ClipKIT kpic-smart-gap	X		S14
RAxML	ClipKIT smart-gap	X		S15
RAxML	trimAl automated1	X		S16
RAxML	trimAl gappyout		X	S17
RAxML	trimAl strict		X	S18
RAxML	original alignment	X		S19

Topology A: Ochrophyta plastids are placed with Cyanidiales; topology B: Ochrophyta plastids branch between Cyanidiales and Proteorhodophytina/Eurhodophytina alongside Cryptophyta/Haptophyta clade (Fig. 1).

^a^Statistics for alignment trimming strategies are provided in supplementary table S1, Supplementary Material online.

^b^CAT model.

Notably, we observed a link between the tree topologies and supermatrix length. Longer supermatrices, with a higher number of variable and parsimony-informative sites, predominantly yielded trees supporting ochrophytes’ grouping with Cyanidiales (9 out of 14 trimming strategies). In contrast, shorter supermatrices favored the alternative topology (5 out of 14 trimming strategies). The untrimmed supermatrix produced both topologies depending on the software: in the RAxML tree, ochrophytes clustered with Cyanidiales (topology A), while in the IQ-TREE phylogeny, they branched between Cyanidiales and Proteorhodophytina/Eurhodophytina alongside Cryptophyta/Haptophyta (topology B) (Table 1, supplementary figs. S11 and S19, Supplementary Material online). Irrespective of ochrophytes’ placement (topology A vs. topology B), our results suggest two separate secondary red alga endosymbioses in Chromalveolata lineages: one driving photosynthesis in the Cryptophyta/Haptophyta clade and the other in Ochrophytes. Importantly, tests of topologies assuming the monophyly of cryptophytes, haptophytes, and ochrophytes, the number of amino acid sites, and testing of individual partitions also supported the model for two red alga plastid transfers to chromalveolates as well as topology A (supplementary fig. S22 and tables S5 to S8, Supplementary Material online).

In our phylogenies, alveolates, specifically colpodellids (*Chromerida* sp. RM11 and *Chromera velia*) and dinoflagellates (*Durinskia baltica* and *Kryptoperidinium foliaceum*), are placed separately within ochrophytes. Colpodellids form a sister clade with Eustigmatophyceae (bootstrap range: 83% to 99% and mean 95% and posterior probabilities 1), while dinoflagellates branch among *Nitzschia*, *Cylindrotheca*, and *Pseudo-nitzschia* species (bootstraps 100% and posterior probabilities 1). Importantly, dinoflagellates are not monophyletic; *D. baltica* is grouped with *Nitzschia palea*, and *K. foliaceum* is their sister, suggesting independent ochrophyte (diatom) endosymbioses in these lineages. Additionally, a dinoflagellate, *Karlodinium veneficum*, clusters with haptophytes (bootstraps 100% and posterior probabilities 1) (Fig. 2, supplementary figs. S2 to S20, Supplementary Material online).

### Molecular Clock Analyses

The estimated ages are presented as means of mean dates from the three molecular clocks calculated (supplementary figs. S21, S23 and S24 and tables S4, S9 and S10 Supplementary Material online, Table 2). Based on our chronograms, the red alga lineage emerged during the Paleoproterozoic Era ∼1.92 billion years ago (Bya). The estimated age for the crown group of red algae is ∼1.75 Bya, corresponding to the divergence of Cyanidiales from the clade comprising Proteorhodophytina and Eurhodophytina (Fig. 2, supplementary figs. S2 and S3, Supplementary Material online).

**Table 2:** Molecular clock estimates for key nodes in million years

Node number and node name	Beast exponential		Beast log normal		Mrbayes IGR		Age mean^a^	Age ranges^b^
	Mean	95% HPD	Mean	95% HPD	Mean	95% HPD		
114 Divergence of Red algae	1,874	1,800 to 2,018	1,882	1,800 to 2,045	1,998	1,807 to 2,329	1,918	1,800 to 2,329
115 Split of Cyanidiales and Proteo./Eurho.	1,707	1,560 to 1,869	1,741	1,560 to 1,930	1,807	1,560 to 2,076	1,752	1,560 to 2,076
116 Split of Cyanidiales and Ochrophytes	1,598	1,360 to 1,819	1,670	1,463 to 1,888	1,740	1,494 to 2,016	1,669	1,360 to 2,016
117 Crown group Ochrophytes	1,451	1,181 to 1,695	1,539	1,337 to 1,744	1,568	1,320 to 1,849	1,519	1,181 to 1,849
148 Divergence of *K. foliaceum*	70	21 to 135	93	44 to 142	88	38 to 143	84	21 to 143
149 Split of *Durinskia* and *Nitzschia*	18	3 to 47	18	5 to 37	16	5 to 30	17	3 to 47
167 Divergence of Colpodellids	1,179	937 to 1,428	1,233	1,023 to 1,454	1,246	1,008 to 1,503	1,220	937 to 1,503
168 Crown group Colpodelids	896	719 to 1,080	997	788 to 1,205	886	671 to 1,111	926	671 to 1,205
175 Crown group Cyanidiales	928	398 to 1,495	1,239	680 to 1,648	1,407	861 to 1,855	1,191	398 to 1,855
178 Split of Proteo./Eurho and Haptophyta/Cryptophyta	1,498	1,182 to 1,758	1,539	1,273 to 1,803	1,543	1,210 to 1,882	1,527	1,182 to 1,882
179 Crown group Proteo. and Eurho.	1,234	1,048 to 1,482	1,210	1,059 to 1,387	1,232	1,077 to 1,435	1,225	1,048 to 1,482
209 Split of Haptophyta and Cryptophyta	1,101	761 to 1,456	1,157	798 to 1,487	1,290	849 to 1,722	1,183	761 to 1,722
210 Crown group Cryptophyta	625	315 to 939	513	315 to 725	506	294 to 771	548	294 to 939
217 Crown group Haptophyta	932	631 to 1,268	1,020	702 to 1,323	1,171	741 to 1,555	1,041	631 to 1,555
219 Divergence of *K. veneficum*	598	399 to 853	656	442 to 881	754	472 to 1,058	669	399 to 1,058

^a^Calculated as means of mean dates from the three chronograms.

^b^The minimum and maximum values of 95% HPD intervals from the three chronograms.

Our analyses strongly indicate that Ochrophytes acquired their plastids directly from red algae, related to Cyanidiales. This event occurred after plastids of Cyanidiales and Ochrophytes diverged ∼1.67 Bya, but before the estimated age of the crown group Ochrophyta ∼1.52 Bya. Furthermore, we determined that ochrophytes transferred their plastids to colpodellids possibly as early as ∼1.22 Bya, when the colpodellid plastid diverged from Ochrophyta, but no later than ∼926 million years ago (Mya), corresponding to the separation age of studied colpodellids (Fig. 2, supplementary figs. S2 and S3, Supplementary Material online).

The provided chronograms suggest the possibility that Cryptophyta acquired plastids after Ochrophytes; however, the opposite scenario is also plausible due to overlapping estimated time frames. According to our clocks, Cryptophyta and Haptophyta plastids diverged from Proteorhodophytina and Eurhodophytina red algae ∼1.53 Bya, but prior to ∼1.18 Bya, i.e. the separation of cryptophytes and haptophytes (Fig. 2, supplementary figs. S2 and S3, Supplementary Material online).

In our studies, we also investigated plastid transfers to dinoflagellates: *K. veneficum*, *K. foliaceum*, and *D. baltica*. They acquired their photosynthetic organelles through three separate endosymbiotic events: ∼670, ∼84, and 17.5 Mya, respectively (Fig. 2, supplementary figs. S2 and S3, Supplementary Material online).

## Discussion

**Fig. 3.**
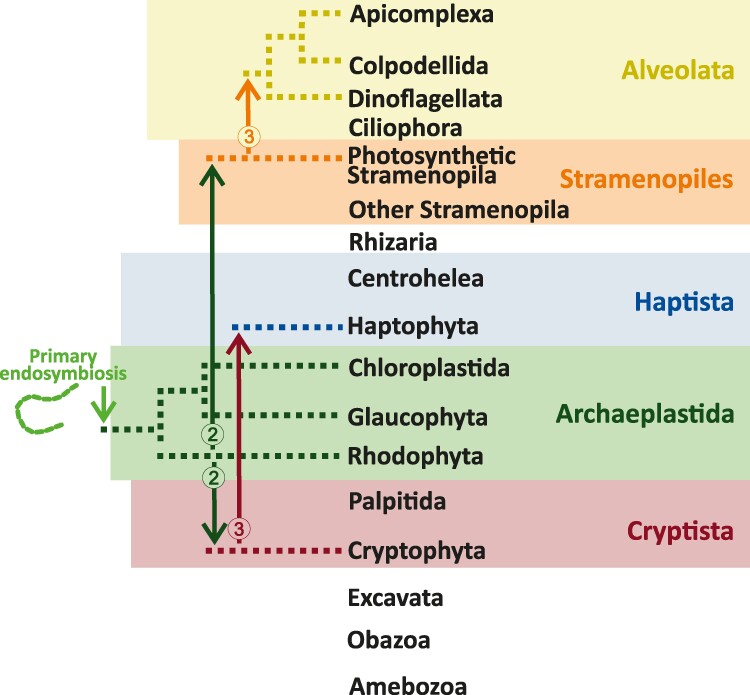
The multiple and serial endosymbiosis model proposed in this study. The arrows indicate plastid transfers, and the numbers in circles correspond to the level of endosymbiosis: 2—secondary and 3—tertiary. The phylogenetic relationships between main eukaryotic lineages (hosts) were based on Strassert et al. (2021). The presented model assumes that cryptophytes donated their plastids to haptophytes rather than haptophytes to cryptophytes since this scenario is more parsimonious considering the presence of a nucleomorph in cryptophyte plastids (a vestigial nucleus of red alga origin between the fourth and the third plastid membranes counting from outside). The alternative scenario would have required haptophytes to carry an endosymbiont with a nucleus and a plastid, which was next donated to cryptophytes, and then, the endosymbiont nucleus was completely reduced in haptophytes but not in cryptophytes.

Our results challenge the Chromalveolata and serial endosymbioses models. The former claims a single red alga endosymbiosis with the ancestor of all Chromalveolata lineages, while the latter claims a single secondary red alga endosymbiosis within cryptophytes, followed by subsequent plastid transfers to other Chromalveolata lineages. Based on host relationships by Strassert et al. (2021), our phylogenies suggest two secondary red alga endosymbioses, one within Cryptophyta and one within Ochrophyta (Fig. 3). Such a possibility has already been mentioned by Dorrell and Bowler (2017) and obtained in Kim et al. (2015) trees. From our phylogenies, we can also infer two independent tertiary endosymbioses: (i) cryptophyte-to-haptophyte and (ii) ochrophyte-to-colpodellid.

Assuming that the colpodellid plastid represents the ancestral alveolate plastid, our results align with the serial endosymbioses models for this particular plastid transfer (Fig. 3, supplementary fig. S1, Supplementary Material online). Notably, our phylogenies and those of Ševčíková et al. (2015) group colpodellids with Eustigmatophyceae. Both lineages lack chlorophyll c, a defining characteristic of Chromalveolata, and instead rely on chlorophyll a, violaxanthin, and β-carotene for capturing light energy (Füssy and Oborník 2017; Amaral et al. 2021). This photosynthetic pigment composition further strengthens the hypothesis that ochrophytes, precisely Eustigmatophyceae, donated their plastid to the ancestor of alveolate plastid–containing lineages.

According to the serial endosymbioses hypotheses, the ochrophyte plastid was replaced in some dinoflagellates by other plastids, e.g. from cryptophytes, other ochrophytes, and haptophytes, and sometimes more than once (Gagat et al. 2014; Bodył 2018). This scenario is also reflected in our phylogenies as *D. baltica* and *K. foliaceum* are separated in our trees by *N. palea*, which indicates two independent ochrophyte endosymbioses in these species. *D. baltica* and *K. foliaceum* represent “dinotoms,” i.e. endosymbiotic consortia of a dinoflagellate and diatom. The former carries a pennate diatom endosymbiont, whereas the latter a centric one (Gagat et al. 2014; Tillmann et al. 2023). Moreover, *K. veneficum* carries a fucoxanthin plastid derived from haptophytes (Gagat et al. 2014).

Our model (Fig. 3) is more parsimonious than previous ones considering the presence of the eubacterial *rpl36* gene copy in the plastomes of cryptophytes and haptophytes (Rice and Palmer 2006). It avoids extra assumptions, e.g. (i) cryptophyte-to-ochrophyte plastid transfer followed by the rpl36 replacement in the cryptophyte plastome and subsequent cryptophyte-to-haptophyte plastid donation or (ii) independent eubacterial *rpl36* acquisitions by both lineages (Bodył, et al. 2009b).

Our model, however, might seem initially less parsimonious than serial endosymbioses models regarding the evolution of protein import into complex plastids. In short, protein import into four-membrane plastids of cryptophytes, haptophytes, and ochrophytes relies on a bipartite presequence: a signal peptide and a transit peptide, and four protein complexes: Sec61, SELMA, Toc, and Tic, located in the fourth (continuous with the host ER), third, second, and first plastid membrane (counting from outside), respectively (Stork et al. 2013). The signal peptide facilitates the crossing of the outermost membrane, whereas the transit peptide enables the crossing of the three remaining ones. A similar system operates in colpodellid/apicomplexan plastids; however, they use vesicular trafficking to connect the host ER with the plastid outermost membrane (Stork et al. 2012). Importantly, the Toc-Tic translocons represent cyanobacteria-host-derived complexes that operate in primary plastids along with transit peptides (Bodył, et al. 2009a; Richardson and Schnell 2020), and Sec61 is an ancient system responsible for protein movement into the ER (Osborne et al. 2005). The only major innovation in complex plastids regarding protein import is SELMA, which evolved from the red alga–derived ERAD complex previously used to export misfolded/aberrant proteins from the ER (Bolte et al. 2011; Felsner et al. 2011; Lau et al. 2016). Under the serial endosymbiosis hypotheses, the ERAD system was adapted only once, while our model requires two independent adaptations. However, unlike the Toc-Tic supercomplex, which represents a chimeric cyanobacterial host innovation, SELMA represents a simple adaptation of a red alga system already used for protein transport. Therefore, we do not consider two independent ERAD adaptations and two subsequent SELMA transfers in two independent tertiary endosymbioses significantly more challenging that one ERAD adaptation and three subsequent SELMA transfers in three independent higher-order endosymbioses. In both scenarios, the same number of the same set of genes is required to be transferred to the host genome.

Comparing our chronograms with clocks calculated by other researchers is challenging because of differences in markers (plastid vs. nuclear), species, calibration points, molecular clock methods, and tree topologies obtained. Nevertheless, meaningful comparisons can still be made by examining the ages of the crown groups of Ochrophyta, Cryptophyta, and Haptophyta from various studies (supplementary table S11, Supplementary Material online). Our estimations for red algae evolution (∼1.92 Bya, crown group ∼1.75 Bya) align with recent molecular clock studies (Strassert et al. 2021; Pietluch et al. 2022) and are consistent with the oldest fossils of red alga discovered (*Rafatazmia chitrakootensis* ∼1.6 Bya) (Bengtson et al. 2017). In our clocks, the crown group of Ochrophyta emerged ∼1.52 Bya, which significantly differs from previous estimations ranging from ∼575 to ∼1.22 Bya. This discrepancy is, however, understandable given the new placement of Ochrophyta in our phylogenies. The estimated age for colpodelids, which is ∼926 Mya, a lineage representing plastid-containing alveolates, is consistent with more recent nuclear marker–based clocks calculated by Parfrey et al. (2011) and Strassert et al. (2021). We inferred the age of the crown group of Cryptophyta as ∼550 Mya and Haptophyta as ∼1,050 Mya. These estimations agree with the lower boundaries for these points calculated by Strassert et al. (2021).

Our results challenge the current view on plastid evolution and suggest that secondary endosymbioses may not be rarer than endosymbioses of a higher order. The two red alga endosymbioses proposed in our scenario provide another example of secondary plastid acquisitions, adding to the already observed two independent endosymbioses of green alga plastids within euglenids and chlorarachniophytes.

## Supplementary Material

evae192_Supplementary_Data

## Data Availability

All data sets used to perform phylogenetic and molecular clock analyses are available at https://zenodo.org/records/11953485.
