# Supplementary material for: A New Model and Dating for the Evolution of Complex Plastids of Red Alga Origin
Source: Genome Biol Evol. 2024 Sep 6;16(9):evae192. doi: 10.1093/gbe/evae192 (PMC11413572; doi:10.1093/gbe/evae192)
Supplement: evae192_Supplementary_Data [file evae192_supplementary_data.pdf]

Supplementary Information for

A New Model and Dating for the Evolution of Complex Plastids of Red Alga Origin

Filip Pietluch[1], Paweł Mackiewicz[1], Kacper Ludwig[1], Przemysław Gagat[1]*

[1]*Department of Bioinformatics and Genomics, Faculty of Biotechnology, University of Wroclaw, 50-383 Wroclaw, Poland*

*corresponding author

Email: przemyslaw.gagat@uwr.edu.pl

This file includes:

Figures S1 to S24

Tables S1 to S29

SI References

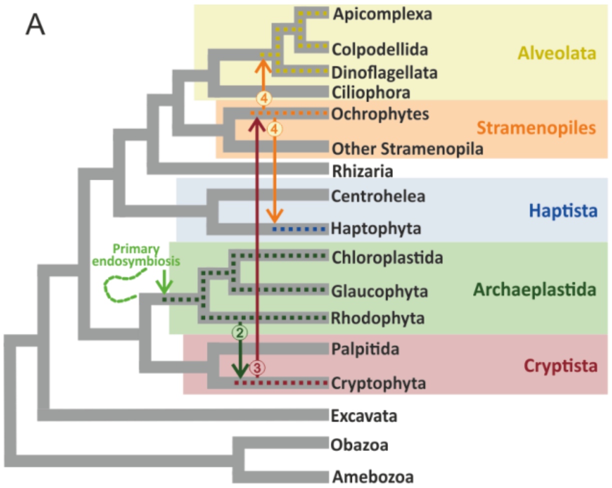

Figure S1. Serial endosymbioses models for complex plastid acquisition. Model A was proposed by Stiller et al. (2014) and Bodył (2018); however the former does not include plastid-containing lineages of Alveolata. Model B visualizes a hypothesis by Bodył et al. (2009). The arrows indicate plastid transfers and the numbers in circles correspond to the level of endosymbiosis: 2 – secondary, 3 – tertiary and 4 - quaternary. The phylogenetic relationships between the main eukaryotic lineages (hosts) are based on Strassert et al. (2021). Both models proposed that only the cryptophyte plastid is of direct red alga descent, i.e. represents a true secondary plastid. According to model A, the cryptophyte plastid was subsequently transferred to some stramenopiles, known as Ochrophyta, in a tertiary endosymbiosis. In turn, ochrophytes passed their plastids to haptists (precisely to Haptophyta) and in Bodył (2018) scenario additionally and independently to the common ancestor of dinoflagellates, perkinsids, colpodellids and apicomplexans. In a quinary endosymbiosis and further endosymbioses (not shown in the figure), a haptophyte plastid replaces the former quaternary ochrophyte-derived plastid in peridinin ‘core’ dinoflagellates but not in their basal lineages, and some peridinin dinoflagellates experience subsequent endosymbiotic plastid switches involving organelles of cryptophytes, ochrophytes and haptophytes (Gagat et al., 2014) (A). According to model B, cryptophytes donated their plastids in two tertiary endosymbioses to: (i) ochrophytes and (ii) haptophytes, and later the latter transferred their plastids to some alveolate lineages (B). The presented models seem complex at first glance but they address a serious problem of the Chromalveola hypothesis, namely that photosynthetic clades are separated on the tree of life by heterotrophic ones (Mansour & Anestis, 2021). The absence of plastids in theses lineages cannot be simply explained by plastid losses because they are extremely rare even though the loss of photosynthesis has occurred multiple times from Archaeplastida to Chromalveolata lineages (Hadariová et al., 2018). The presented serial endosymbioses models restrict plastid losses to only selected parasitic lineages such as apicomplexans (*Cryptosporidium* and gregarines) and dinoflagellates (*Hematodinium*) (Sibbald & Archibald, 2020).

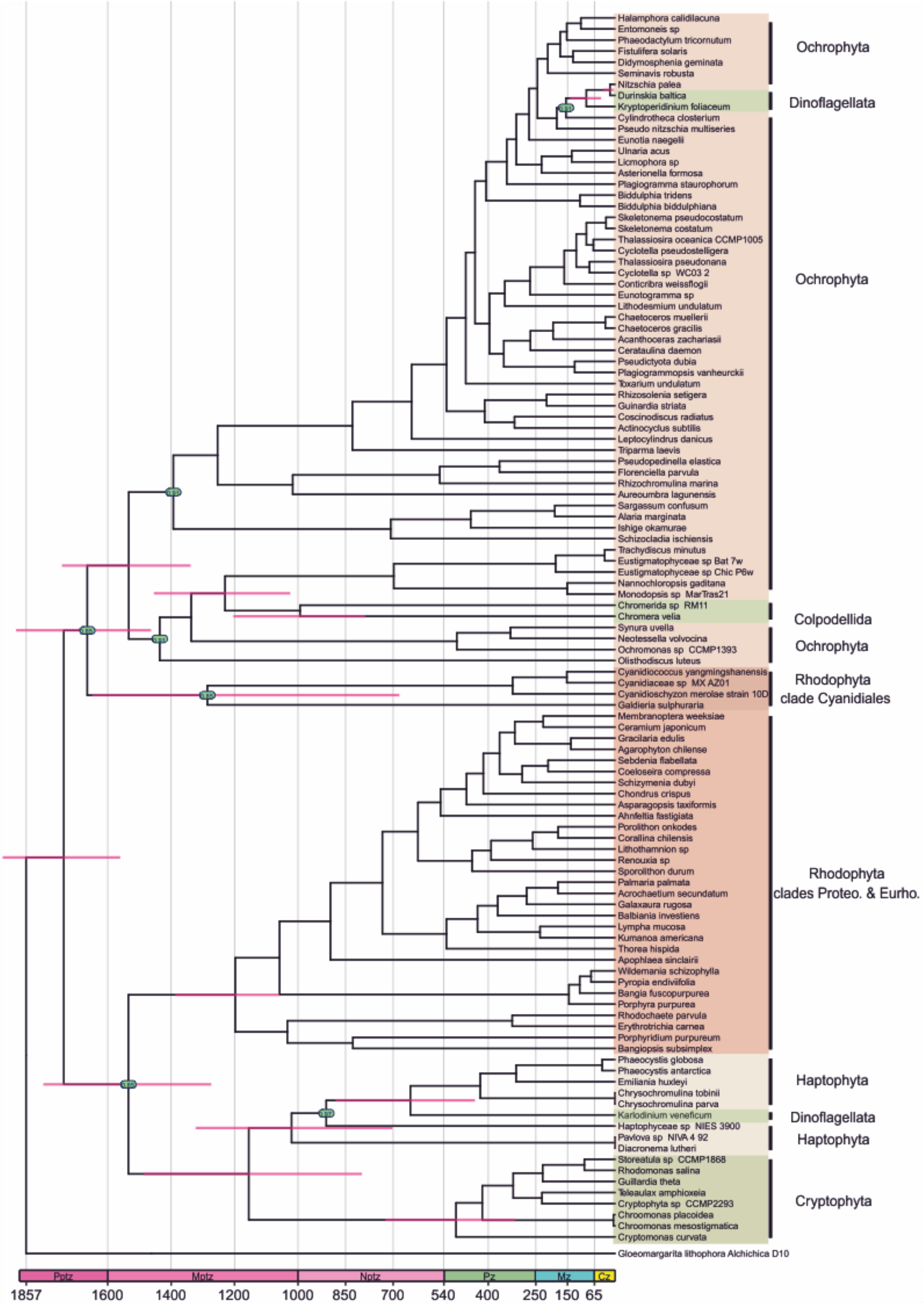

Figure S2. Time-calibrated phylogeny of red alga and red alga-derived plastids. The tree was inferred with Beast (Bouckaert et al., 2014) using lognormal model, based on alignment produced by ClipKIT smart-gap algorithm (Steenwyk et al., 2020) along with substitution models provided in Table S2, and calibrated with constraints (numbers in white circles) listed in Table S3. Node numbers to help reading time estimates are in Figure S23 and the estimates in Table S9. Nodes with posterior probability lower than one are marked with green circles. Shortcuts on the geological time scale for Eras: Cz – Cenozoic, Mz – Mesozoic, Pz – Paleozoic, Nptz – Neoproterozoic, Mptz – Mesoproterozoic, Pptz – Paleoproterozoic.

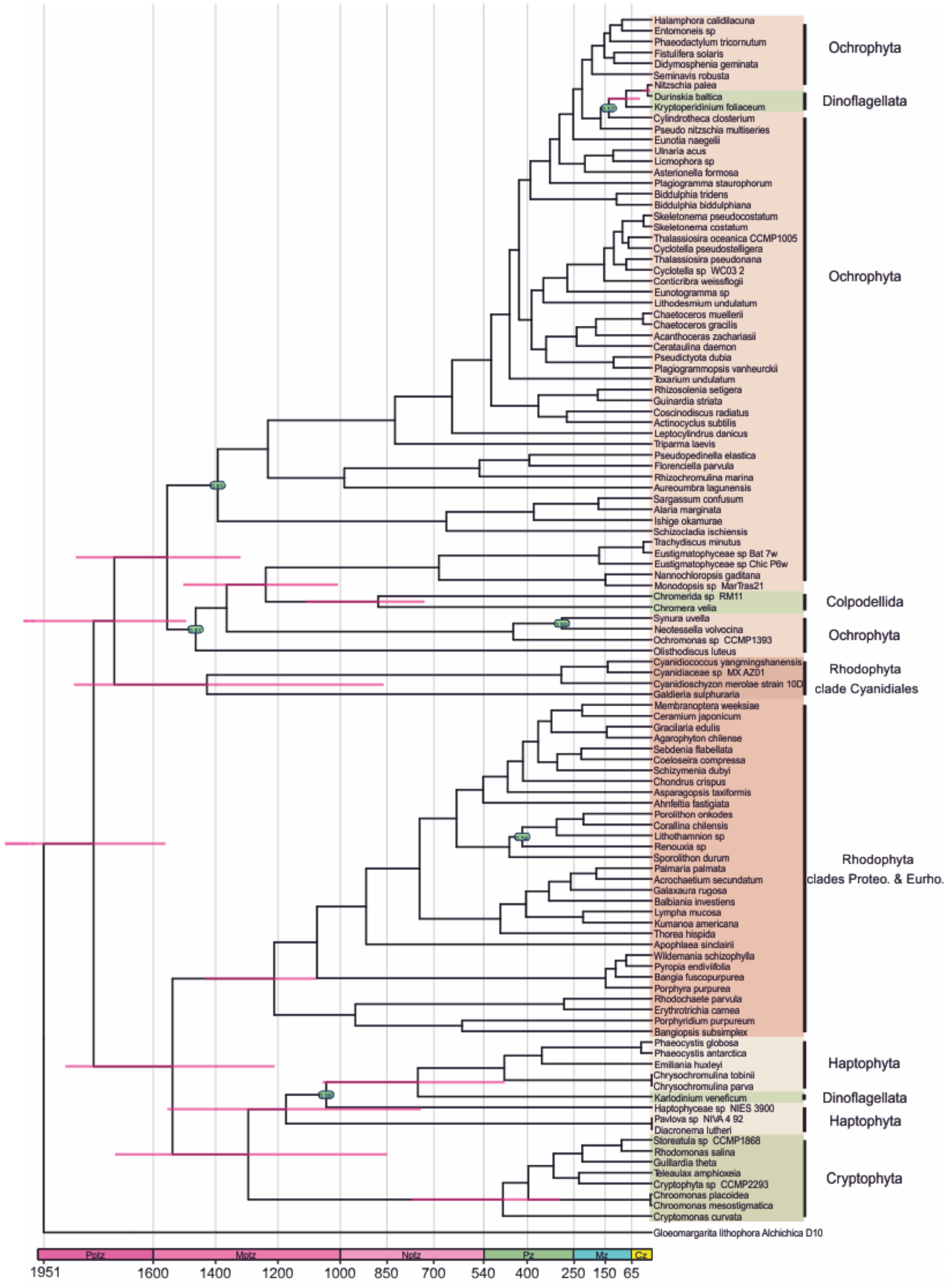

Figure S3. Time-calibrated phylogeny of red alga and red alga-derived plastids. The tree was inferred with MrBayes (Ronquist et al., 2012) using IGR model, based on alignment produced by ClipKIT smart-gap algorithm (Steenwyk et al., 2020) along with substitution models provided in Table S8, calibrated with constraints (numbers in white circles) listed in Table S3 and rooted in Gloeomargarita lithophora Alchichica D10. Node numbers to help reading time estimates are in Figure S24 and the estimates in Table S10. Nodes with posterior probability lower than one are marked with green circles. Shortcuts on the geological time scale for Eras: Cz – Cenozoic, Mz – Mesozoic, Pz – Paleozoic, Nptz – Neoproterozoic, Mptz – Mesoproterozoic, Pptz – Paleoproterozoic.

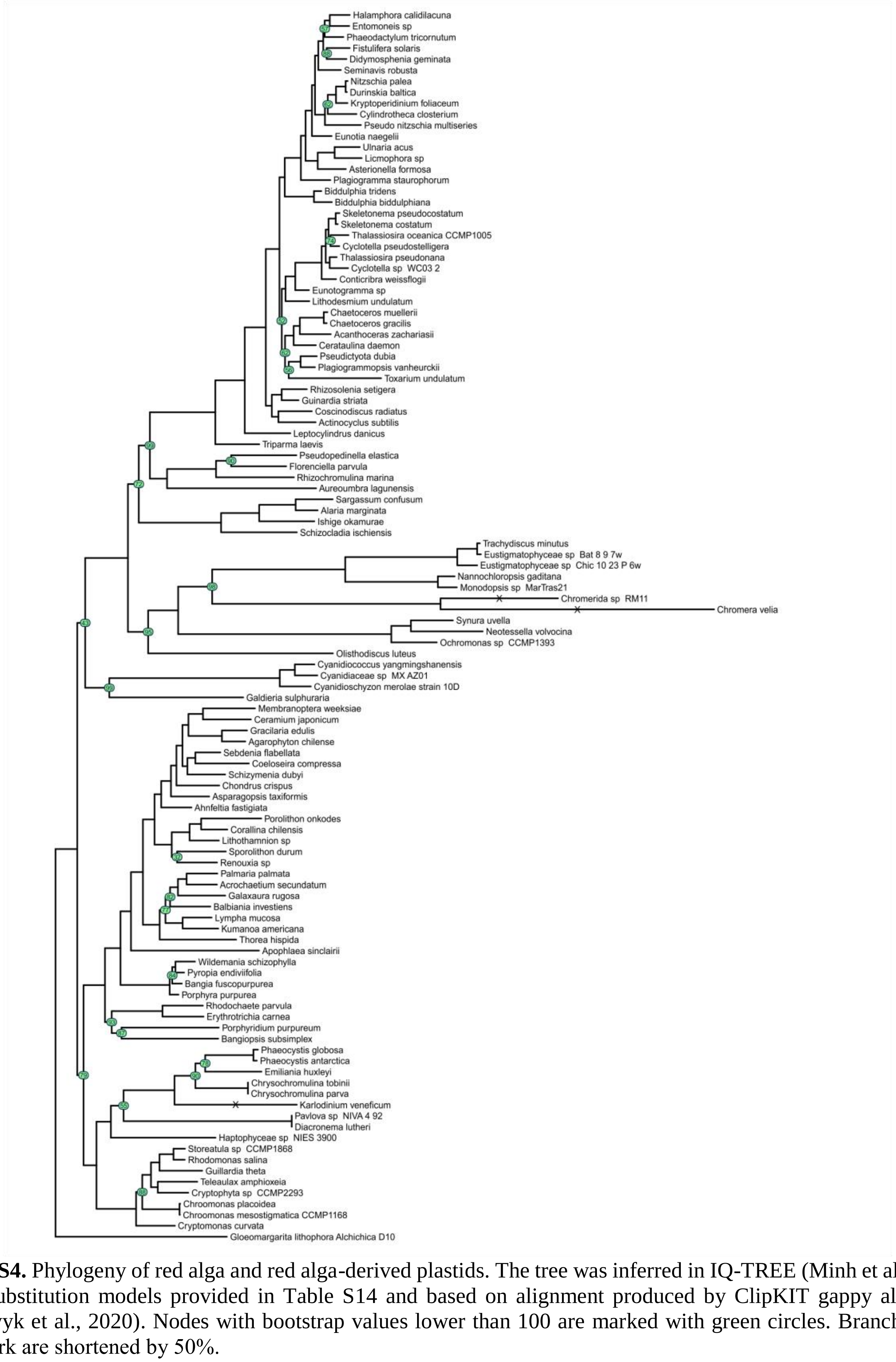

Figure S4. Phylogeny of red alga and red alga-derived plastids. The tree was inferred in IQ-TREE (Minh et al., 2020) using substitution models provided in Table S14 and based on alignment produced by ClipKIT gappy algorithm (Steenwyk et al., 2020). Nodes with bootstrap values lower than 100 are marked with green circles. Branches with "X" mark are shortened by 50%.

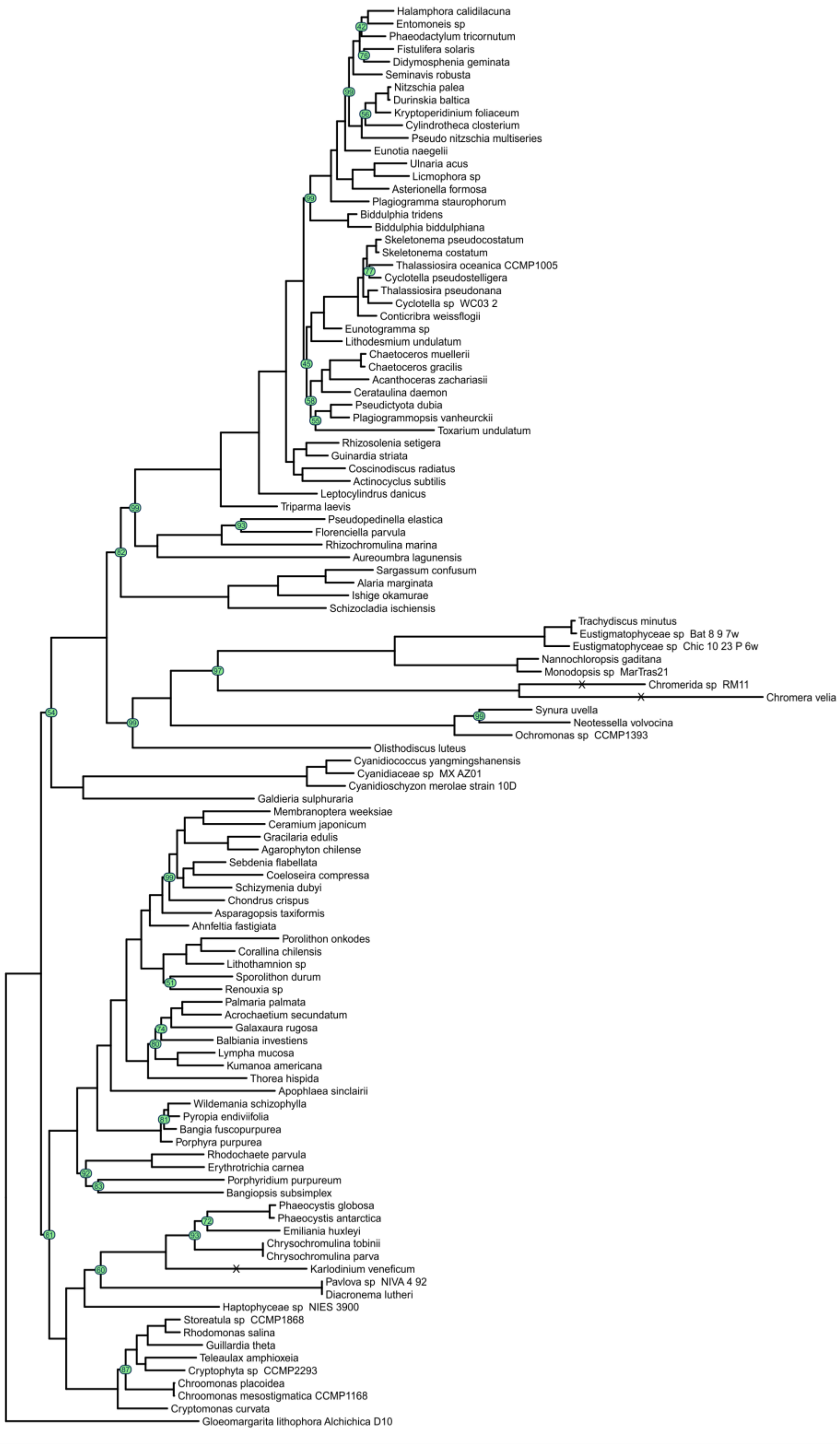

Figure S5. Phylogeny of red alga and red alga-derived plastids. The tree was inferred in IQ-TREE (Minh et al., 2020) using substitution models provided in Table S15 and based on alignment produced by ClipKIT kpic algorithm (Steenwyk et al., 2020). Nodes with bootstrap values lower than 100 are marked with green circles. Branches with "X" mark are shortened by 50%.

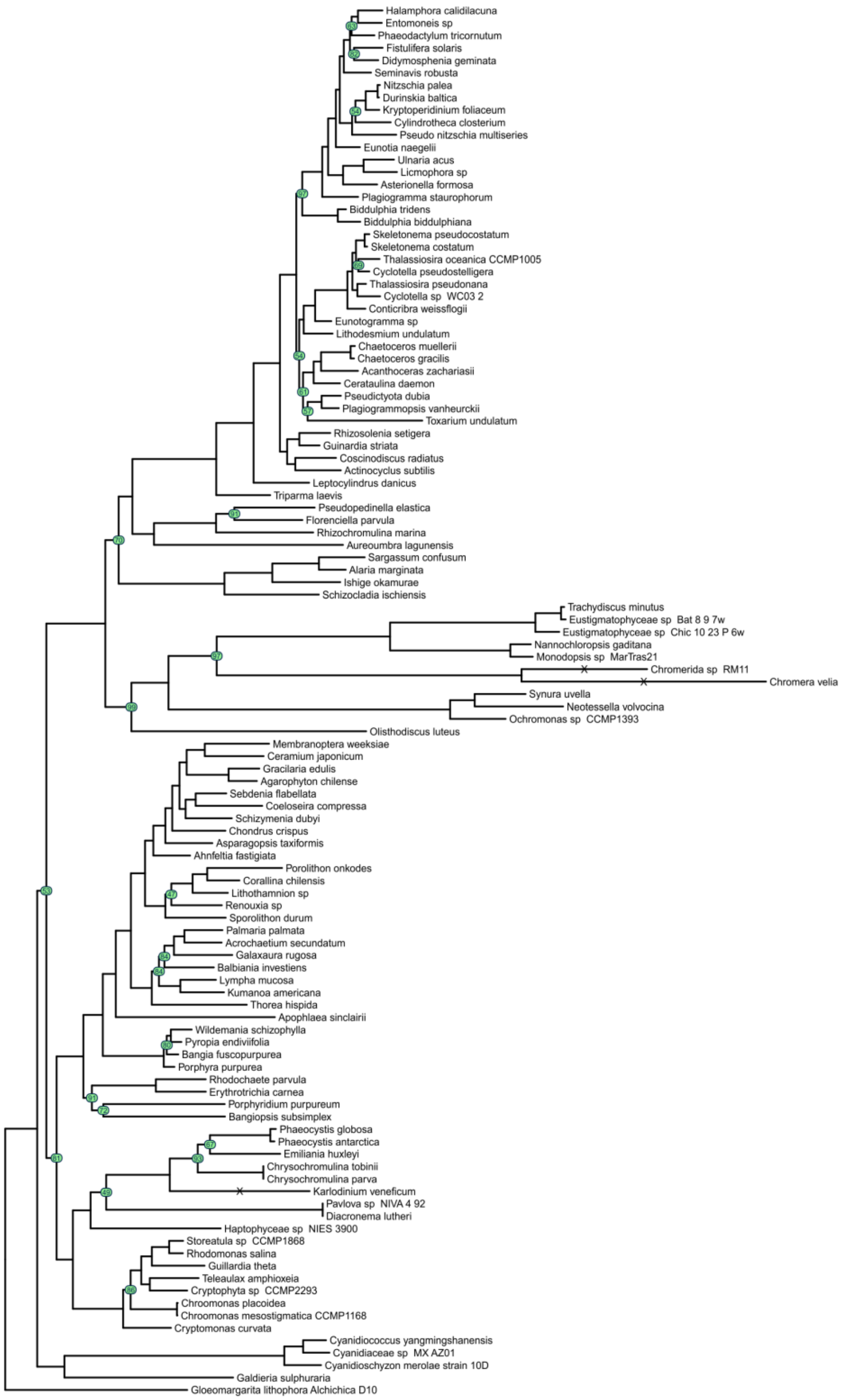

Figure S6. Phylogeny of red alga and red alga-derived plastids. The tree was inferred in IQ-TREE (Minh et al., 2020) using substitution models provided in Table S16 and based on alignment produced by ClipKIT kpic-smart-gap algorithm (Steenwyk et al., 2020). Nodes with bootstrap values lower than 100 are marked with green circles. Branches with “X” mark are shortened by 50%.

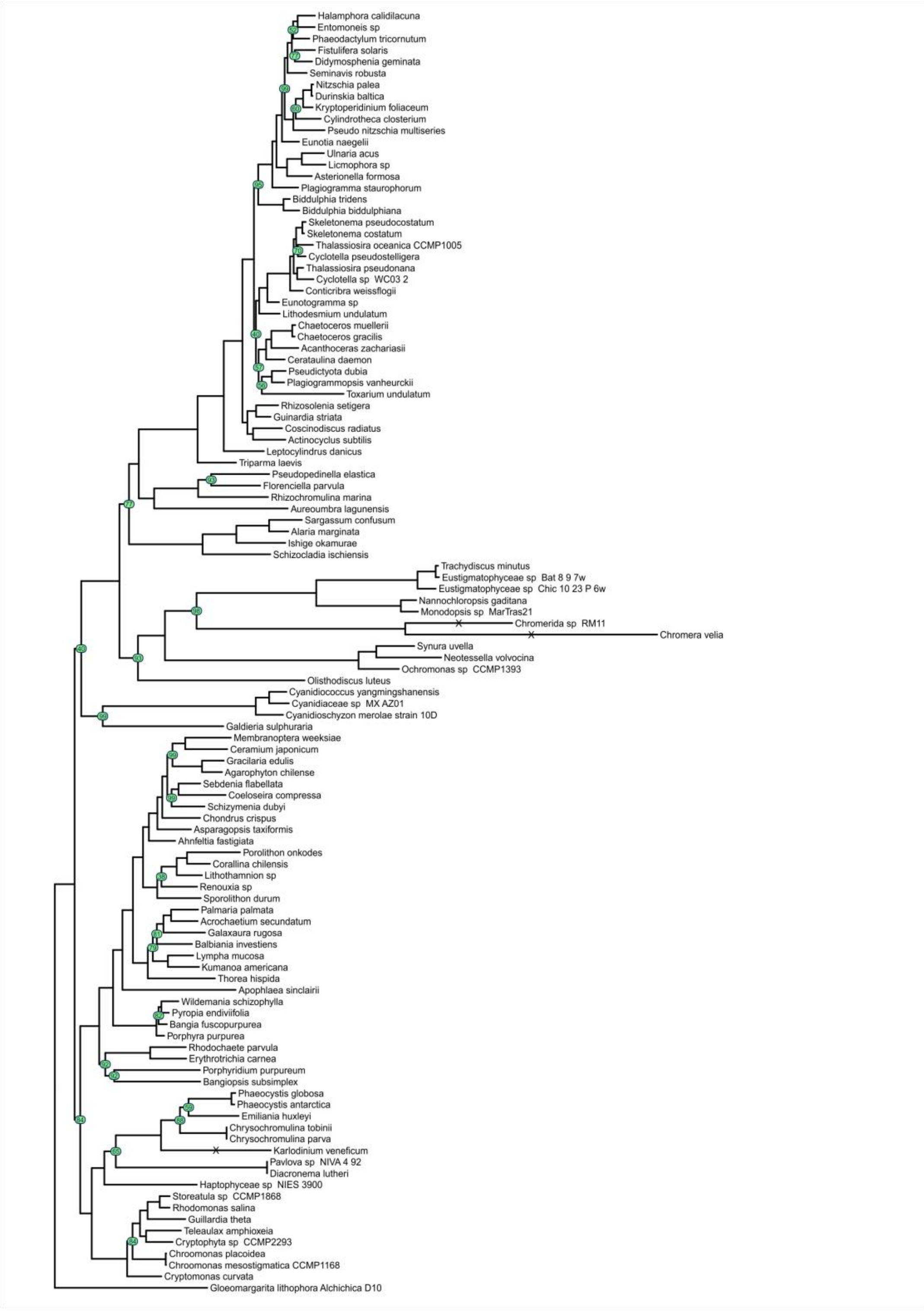

Figure S7. Phylogeny of red alga and red alga-derived plastids. The tree was inferred in IQ-TREE (Minh et al., 2020) using substitution models provided in Table S17 and based on alignment produced by ClipKIT smart-gap algorithm algorithm (Steenwyk et al., 2020). Nodes with bootstrap values lower than 100 are marked with green circles. Branches with "X" mark are shortened by 50%.

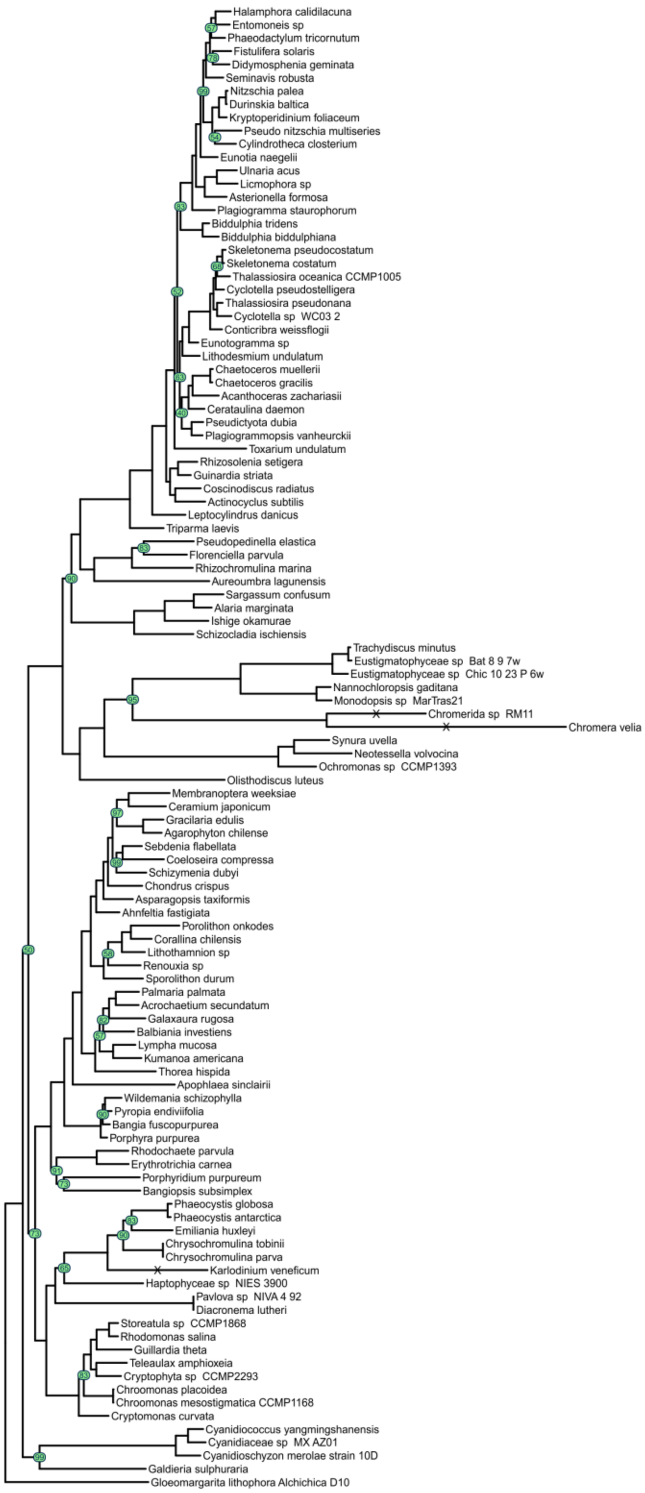

Figure S8. Phylogeny of red alga and red alga-derived plastids. The tree was inferred in IQ-TREE (Minh et al., 2020) using substitution models provided in Table S18 and based on alignment produced by trimAl automated1 algorithm (Capella-Gutiérrez et al., 2009). Nodes with bootstrap values lower than 100 are marked with green circles. Branches with “X” mark are shortened by 50%.

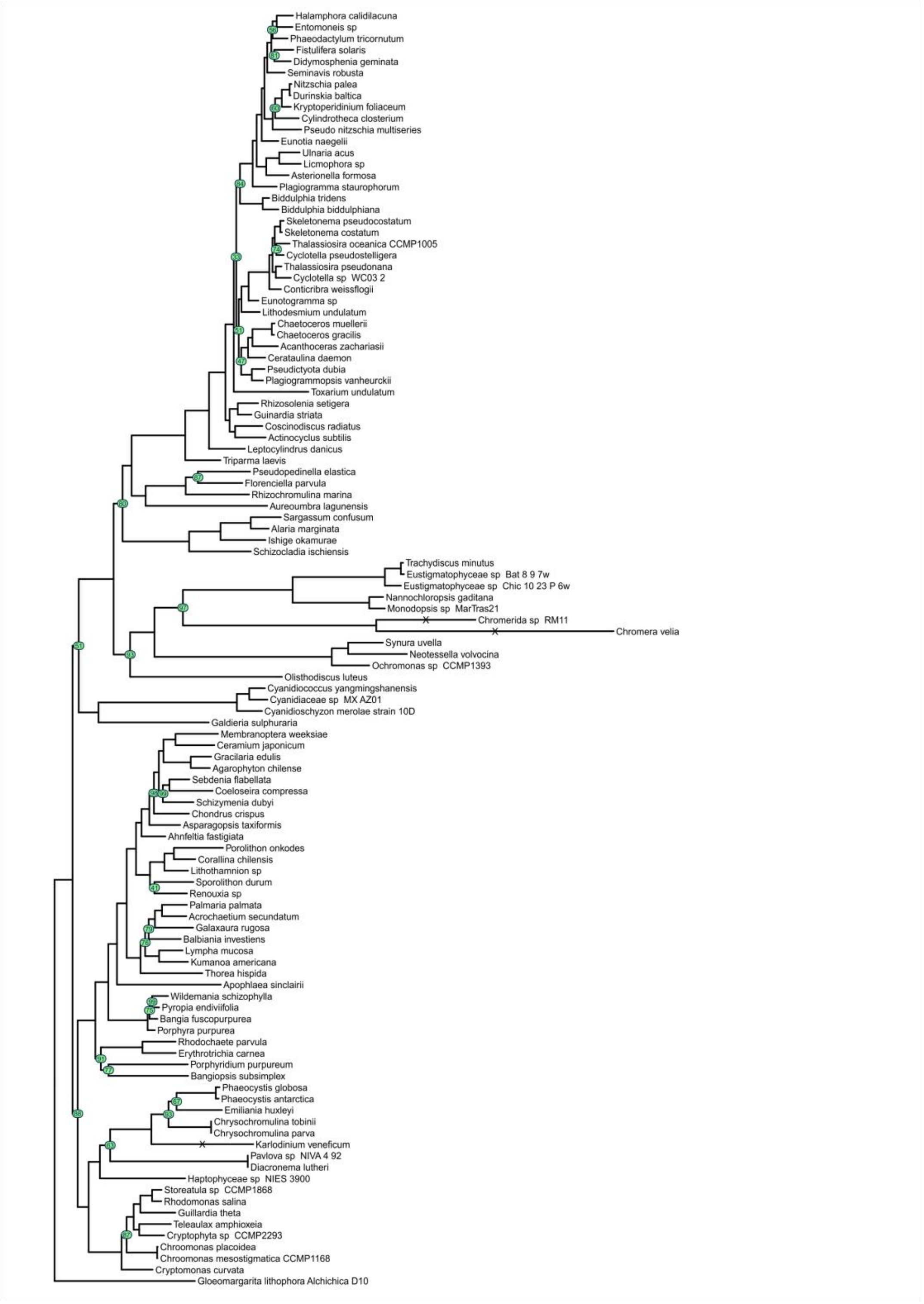

Figure S9. Phylogeny of red alga and red alga-derived plastids. The tree was inferred in IQ-TREE (Minh et al., 2020) using substitution models provided in Table S19 and based on alignment produced by trimAl gappyout algorithm (Capella-Gutiérrez et al., 2009). Nodes with bootstrap values lower than 100 are marked with green circles. Branches with "X" mark are shortened by 50%.

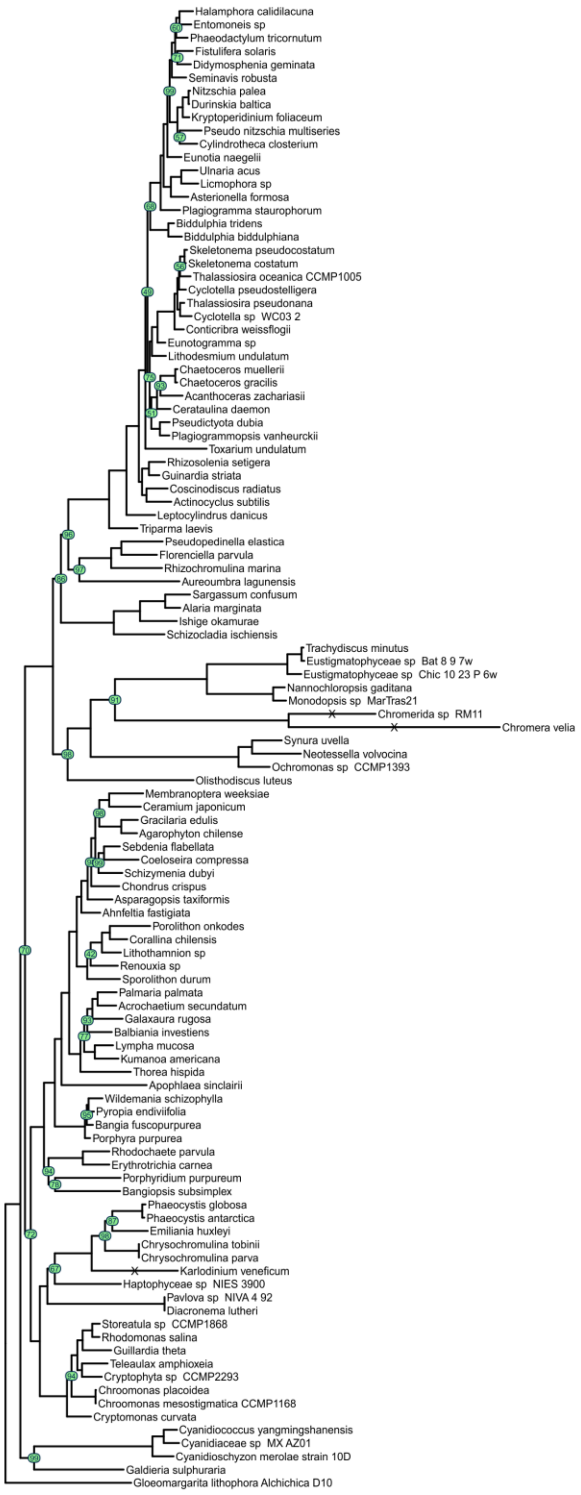

Figure S10. Phylogeny of red alga and red alga-derived plastids. The tree was inferred in IQ-TREE (Minh et al., 2020) using substitution models provided in Table S20 and based on alignment produced by trimAl strict algorithm (Capella-Gutiérrez et al., 2009). Nodes with bootstrap values lower than 100 are marked with green circles. Branches with "X" mark are shortened by 50%.

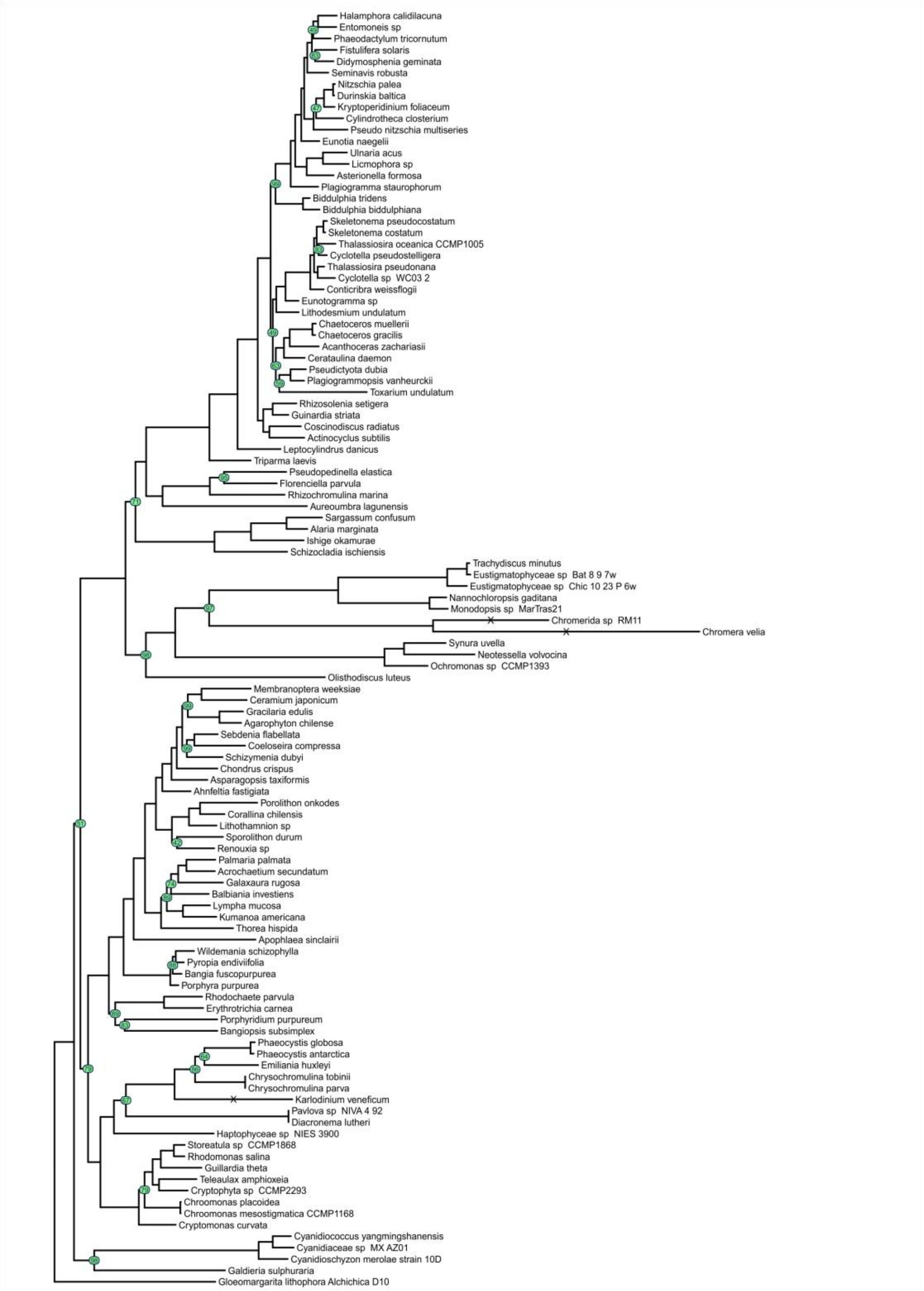

Figure S11. Phylogeny of red alga and red alga-derived plastids. The tree was inferred in IQ-TREE (Minh et al., 2020) using substitution models provided in Table S21, based on original (untrimmed) alignment. Nodes with bootstrap values lower than 100 are marked with green circles. Branches with "X" mark are shortened by 50%.

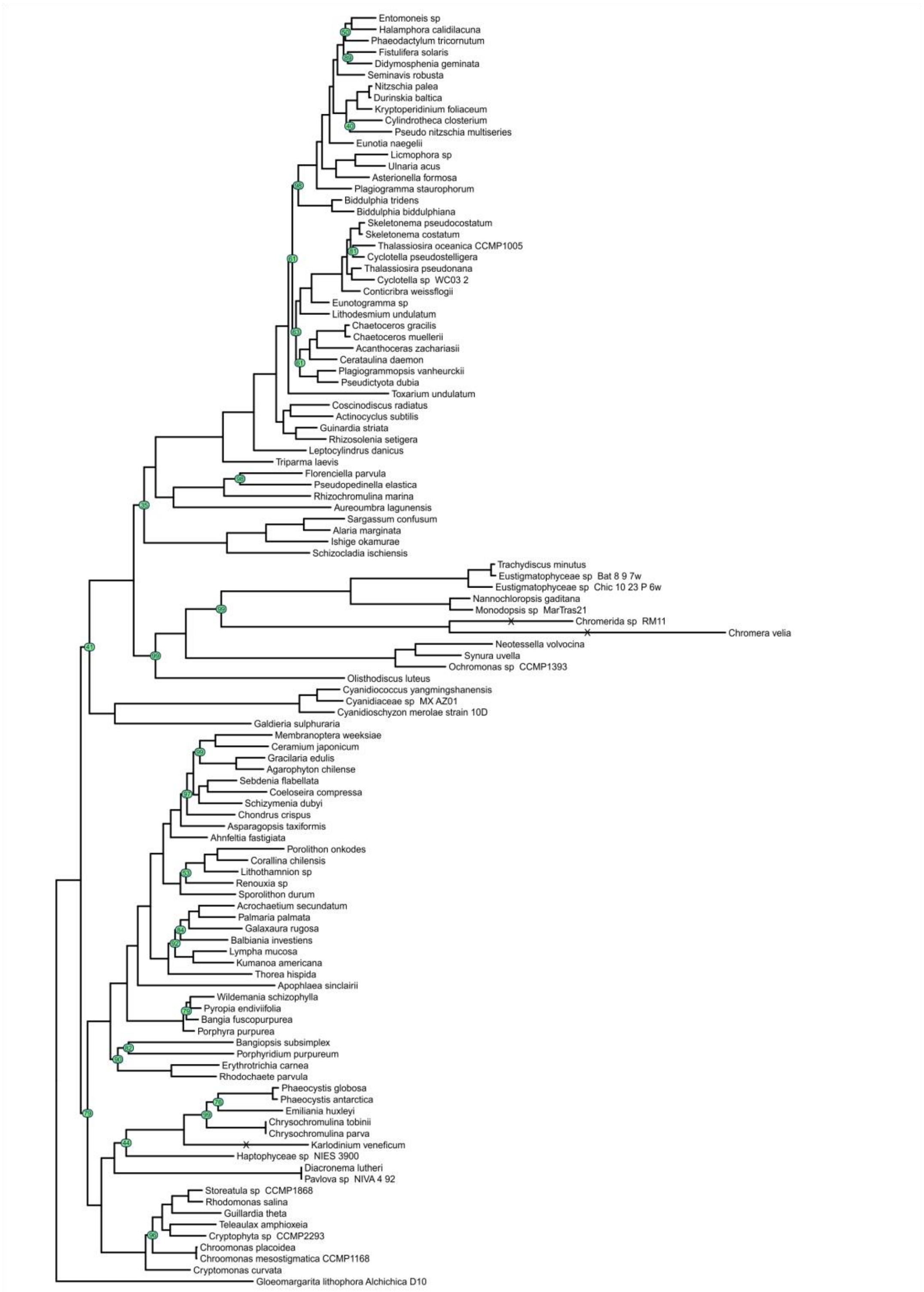

Figure S12. Phylogeny of red alga and red alga-derived plastids. The tree was inferred in RAxML (Stamatakis, 2014) using substitution models provided in Table S22, based on alignment produced by ClipKIT gappy algorithm (Steenwyk et al., 2020) and rooted in *Gloeomargarita lithophora* Alchichica D10. Nodes with bootstrap values lower than 100 are marked with green circles. Branches with “X” mark are shortened by 50%.

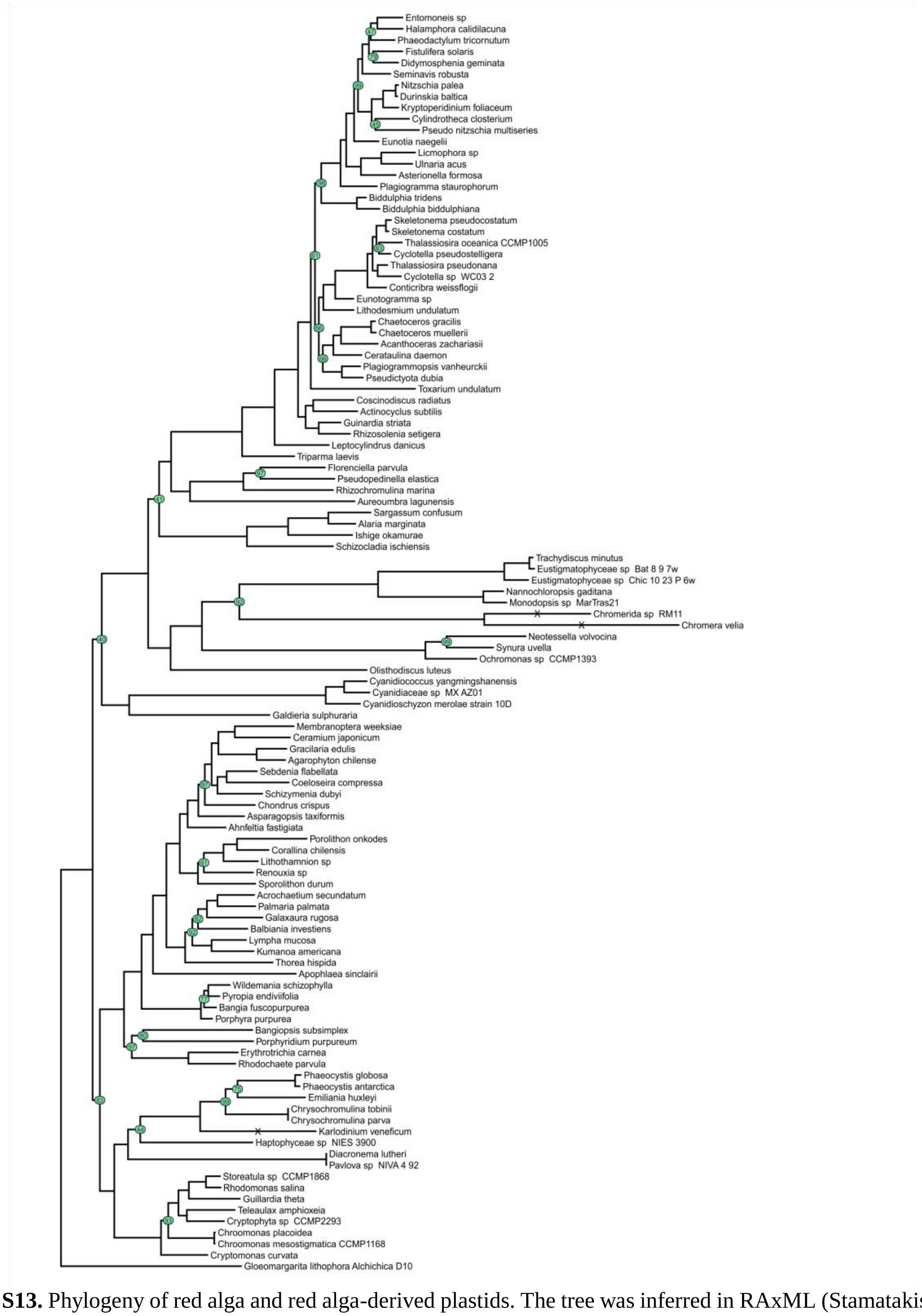

Figure S13. Phylogeny of red alga and red alga-derived plastids. The tree was inferred in RAxML (Stamatakis, 2014) using substitution models provided in Table S23, based on alignment produced by ClipKIT kpic algorithm (Steenwyk et al., 2020) and rooted in *Gloeomargarita lithophora* Alchichica D10. Nodes with bootstrap values lower than 100 are marked with green circles. Branches with "X" mark are shortened by 50%.

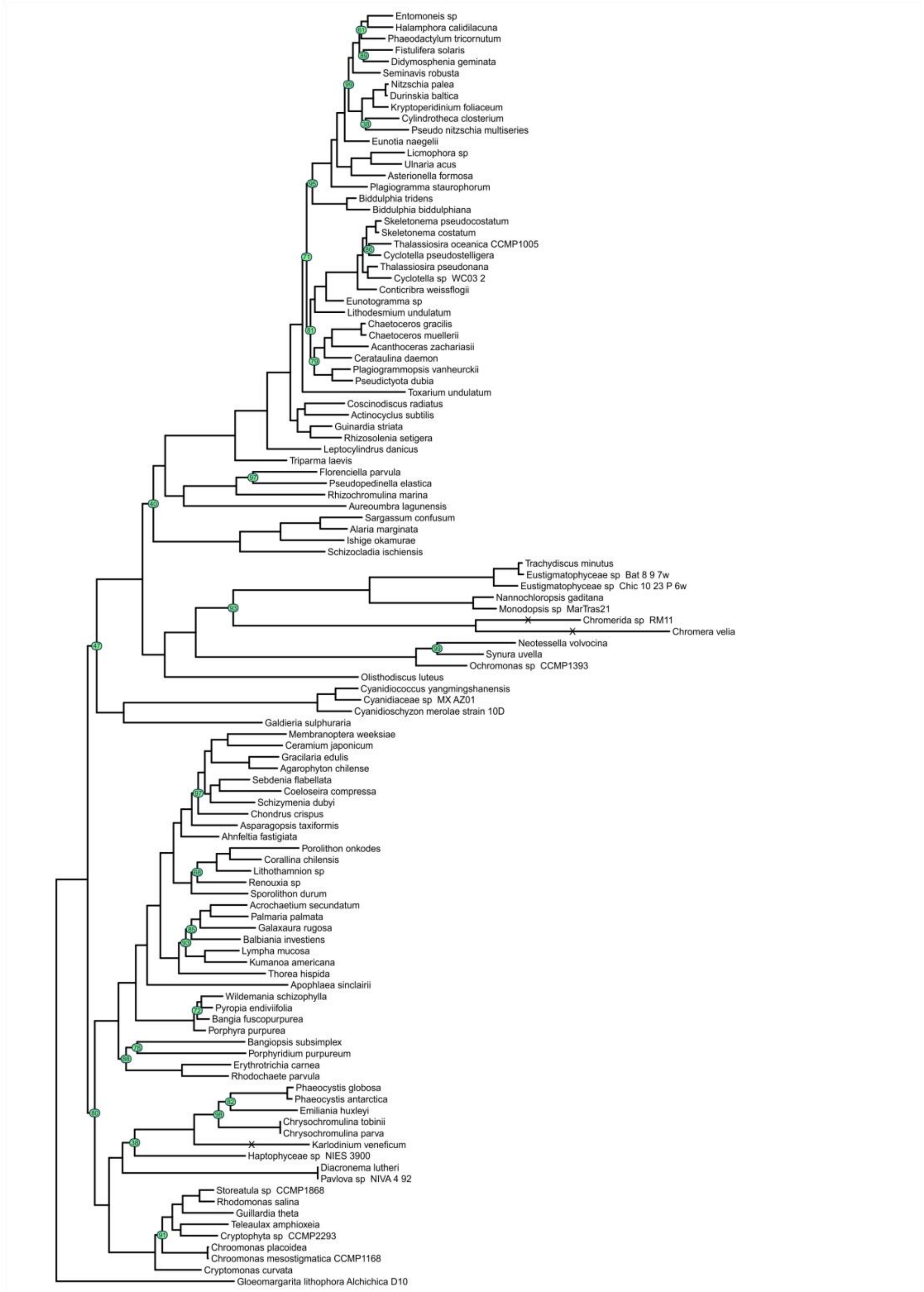

Figure S14. Phylogeny of red alga and red alga-derived plastids. The tree was inferred in RAxML (Stamatakis, 2014) using substitution models provided in Table S24, based on alignment produced by ClipKIT kpic-smart-gap algorithm (Steenwyk et al., 2020) and rooted in *Gloeomargarita lithophora* Alchichica D10. Nodes with bootstrap values lower than 100 are marked with green circles. Branches with "X" mark are shortened by 50%.

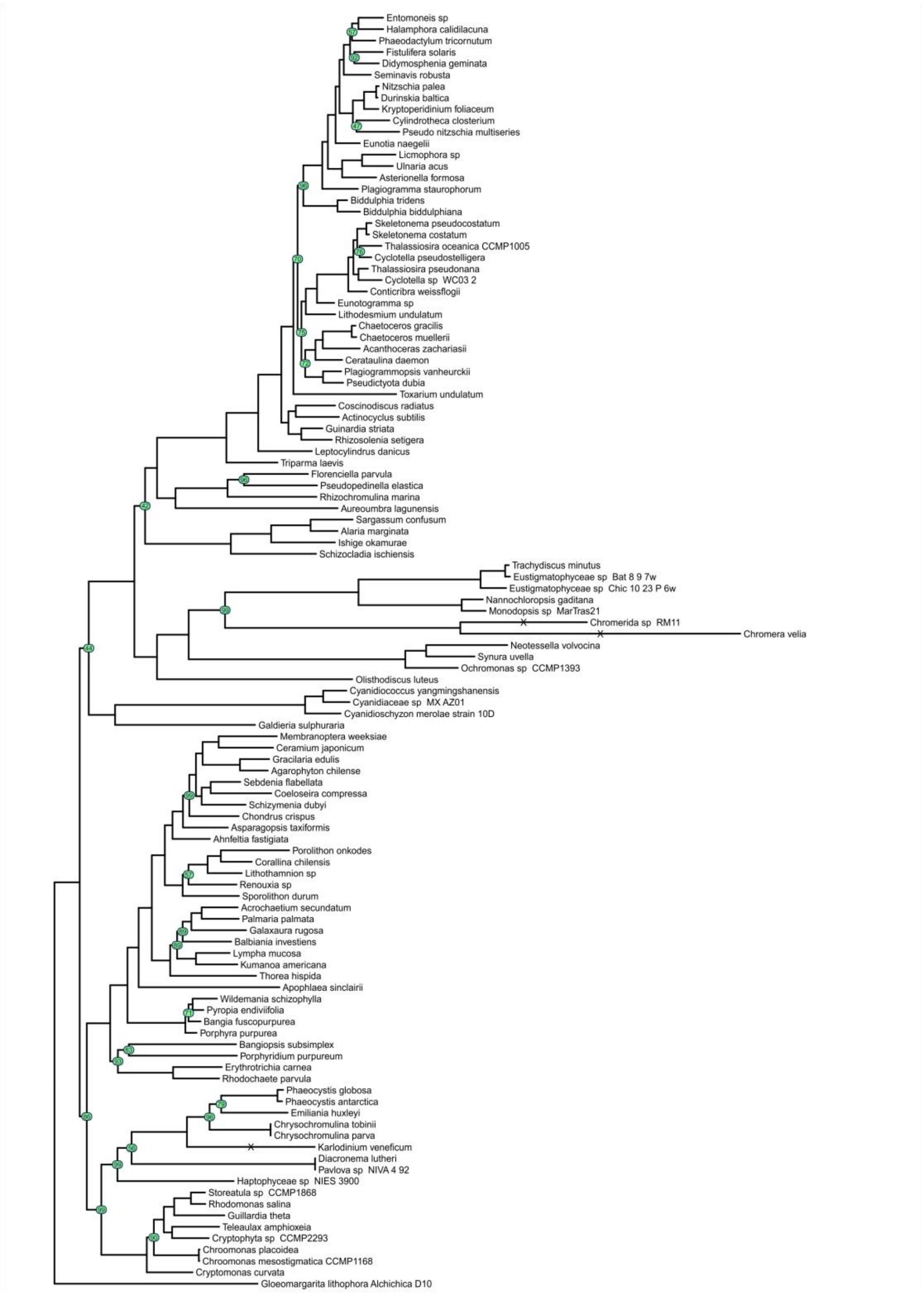

Figure S15. Phylogeny of red alga and red alga-derived plastids. The tree was inferred in RAxML (Stamatakis, 2014) using substitution models provided in Table S25, based on alignment produced by ClipKIT smart-gap algorithm (Steenwyk et al., 2020) and rooted in *Gloeomargarita lithophora* Alchichica D10. Nodes with bootstrap values lower than 100 are marked with green circles. Branches with "X" mark are shortened by 50%.

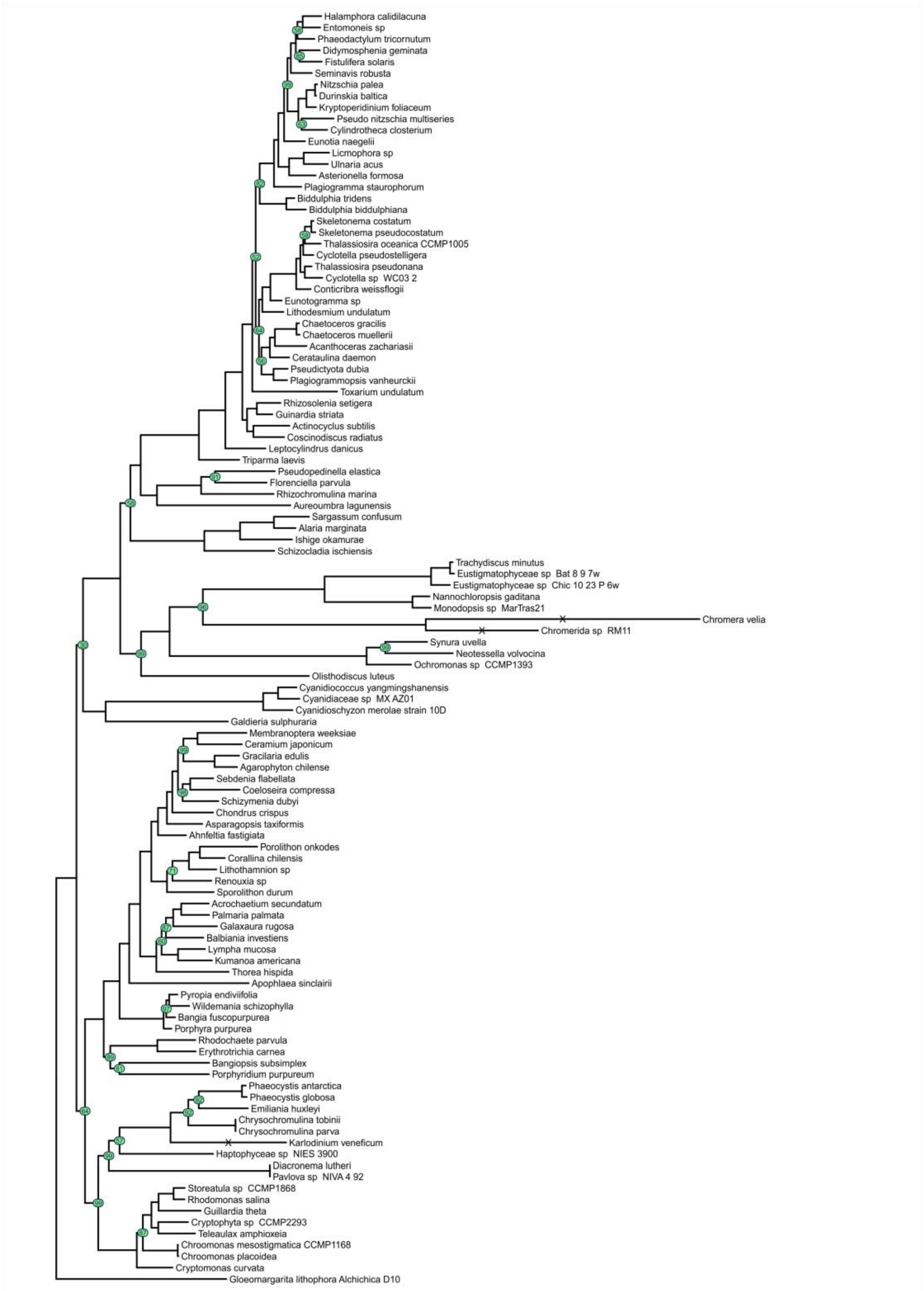

Figure S16. Phylogeny of red alga and red alga-derived plastids. The tree was inferred in RAxML (Stamatakis, 2014) using substitution models provided in Table S26, based on alignment produced by trimAl automated1 algorithm (Capella-Gutiérrez et al., 2009) and rooted in *Gloeomargarita lithophora* Alchichica D10. Nodes with bootstrap values lower than 100 are marked with green circles. Branches with "X" mark are shortened by 50%.

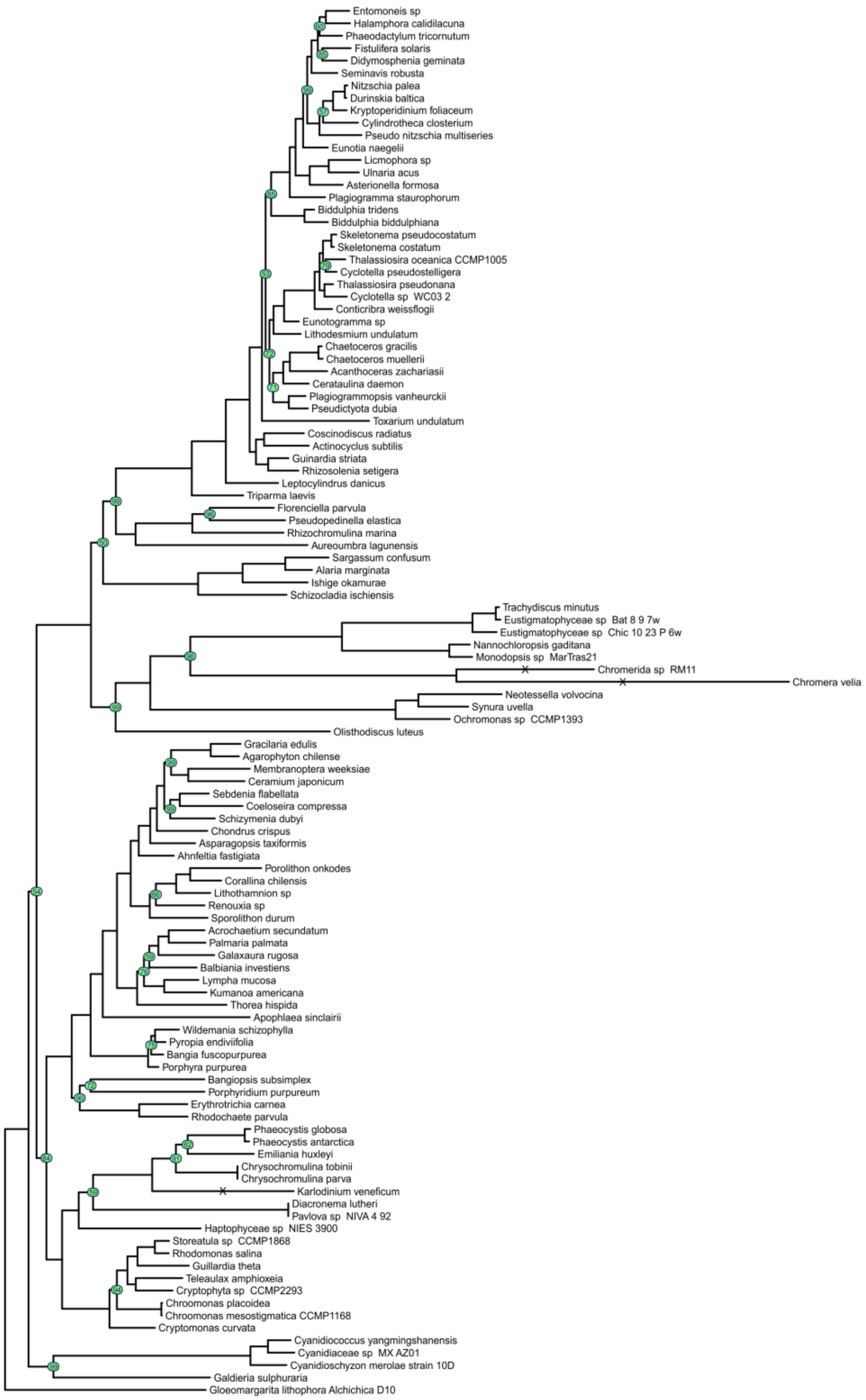

Figure S17. Phylogeny of red alga and red alga-derived plastids. The tree was inferred in RAxML (Stamatakis, 2014) using substitution models provided in Table S25, based on alignment produced by trimAl gappyout algorithm (Capella-Gutiérrez et al., 2009) and rooted in *Gloeomargarita lithophora* Alchichica D10. Nodes with bootstrap values lower than 100 are marked with green circles. Branches with "X" mark are shortened by 50%.

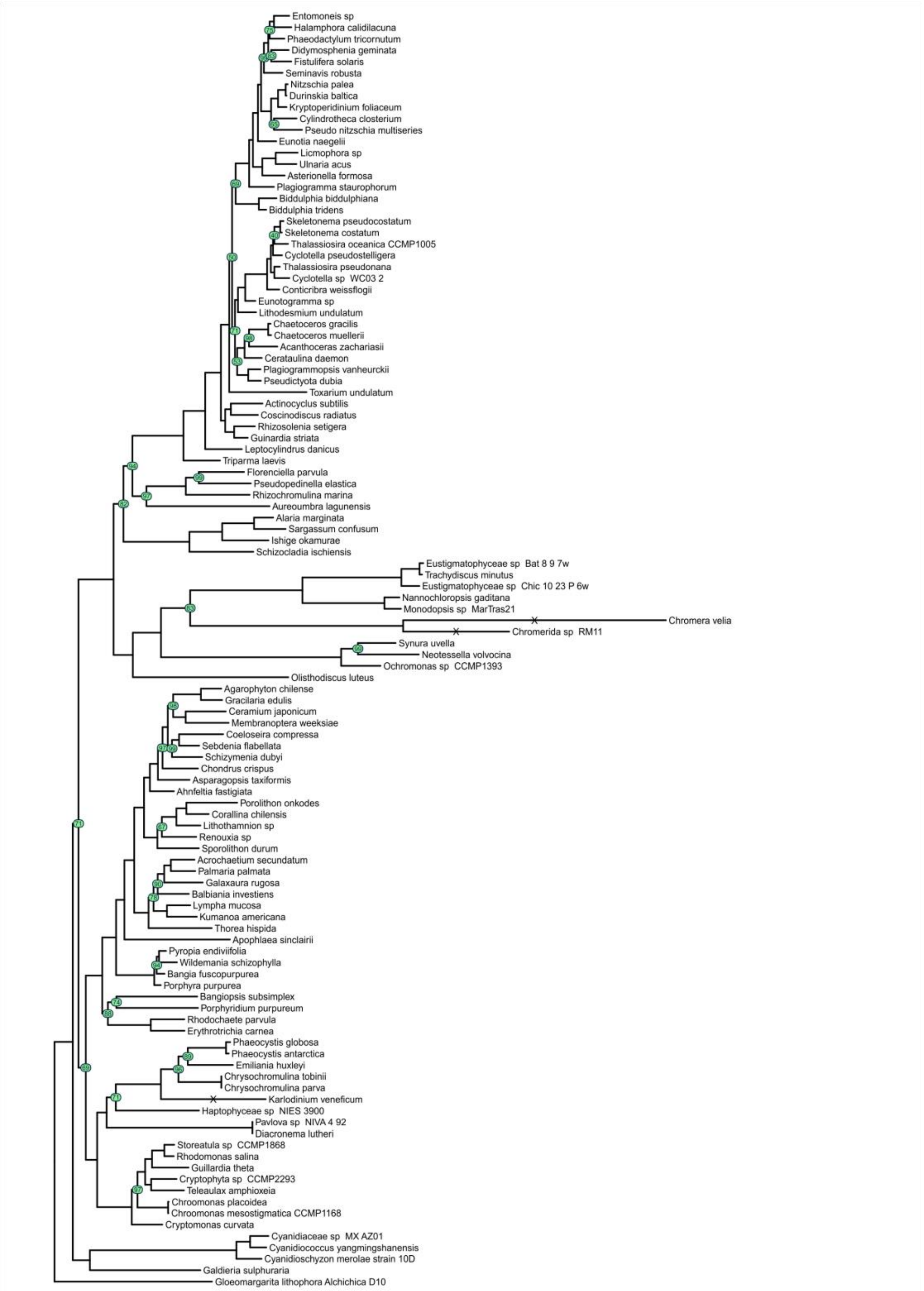

Figure S18. Phylogeny of red alga and red alga-derived plastids. The tree was inferred in RAxML (Stamatakis, 2014) using substitution models provided in Table S28, based on alignment produced by trimAl strict algorithm (Capella-Gutiérrez et al., 2009) and rooted in *Gloeomargarita lithophora* Alchichica D10. Nodes with bootstrap values lower than 100 are marked with green circles. Branches with “X” mark are shortened by 50%.

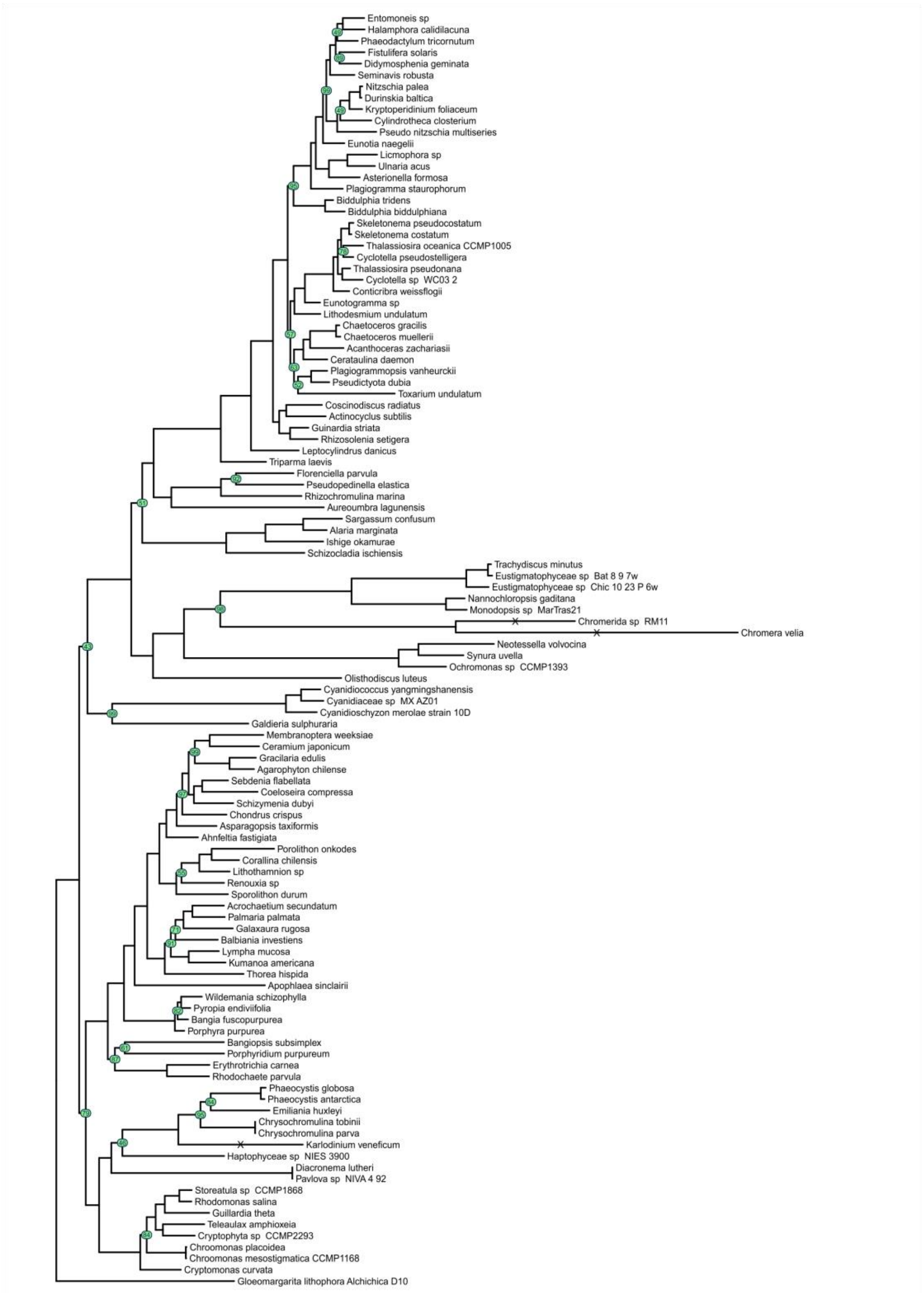

Figure S19. Phylogeny of red alga and red alga-derived plastids. The tree was inferred in RAxML (Stamatakis, 2014) using substitution models provided in Table S29, based on original (untrimmed) alignment and rooted in *Gloeomargarita lithophora* Alchichica D10. Nodes with bootstrap values lower than 100 are marked with green circles. Branches with "X" mark are shortened by 50%.

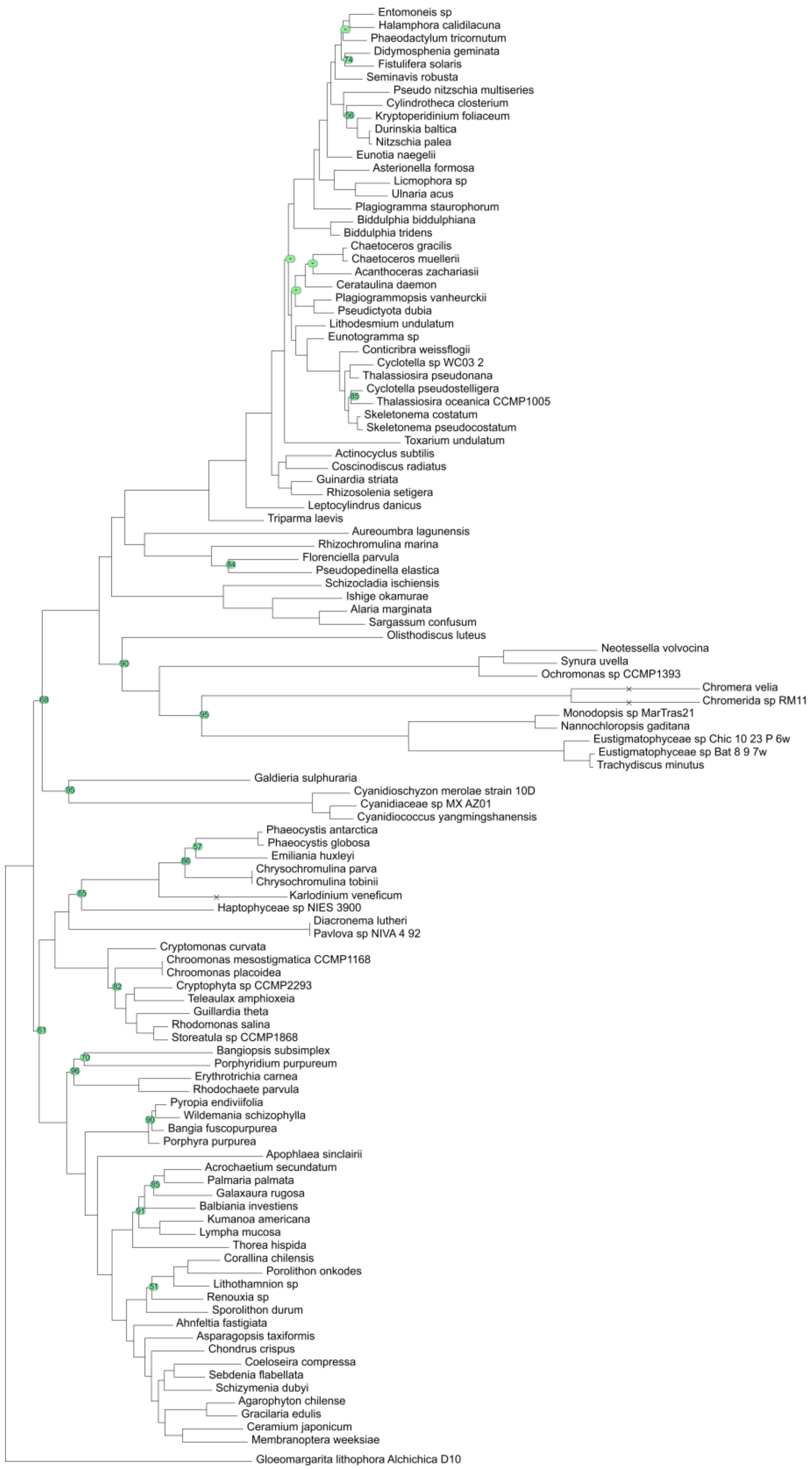

Figure S20. Phylogeny of red alga and red alga-derived plastids. The tree topology was reconstructed in IQ-TREE using CAT model (Minh et al., 2020) and based on alignment produced by ClipKIT smart-gap algorithm (Steenwyk et al., 2020). Nodes with bootstrap values lower than 100 are marked with green circles.

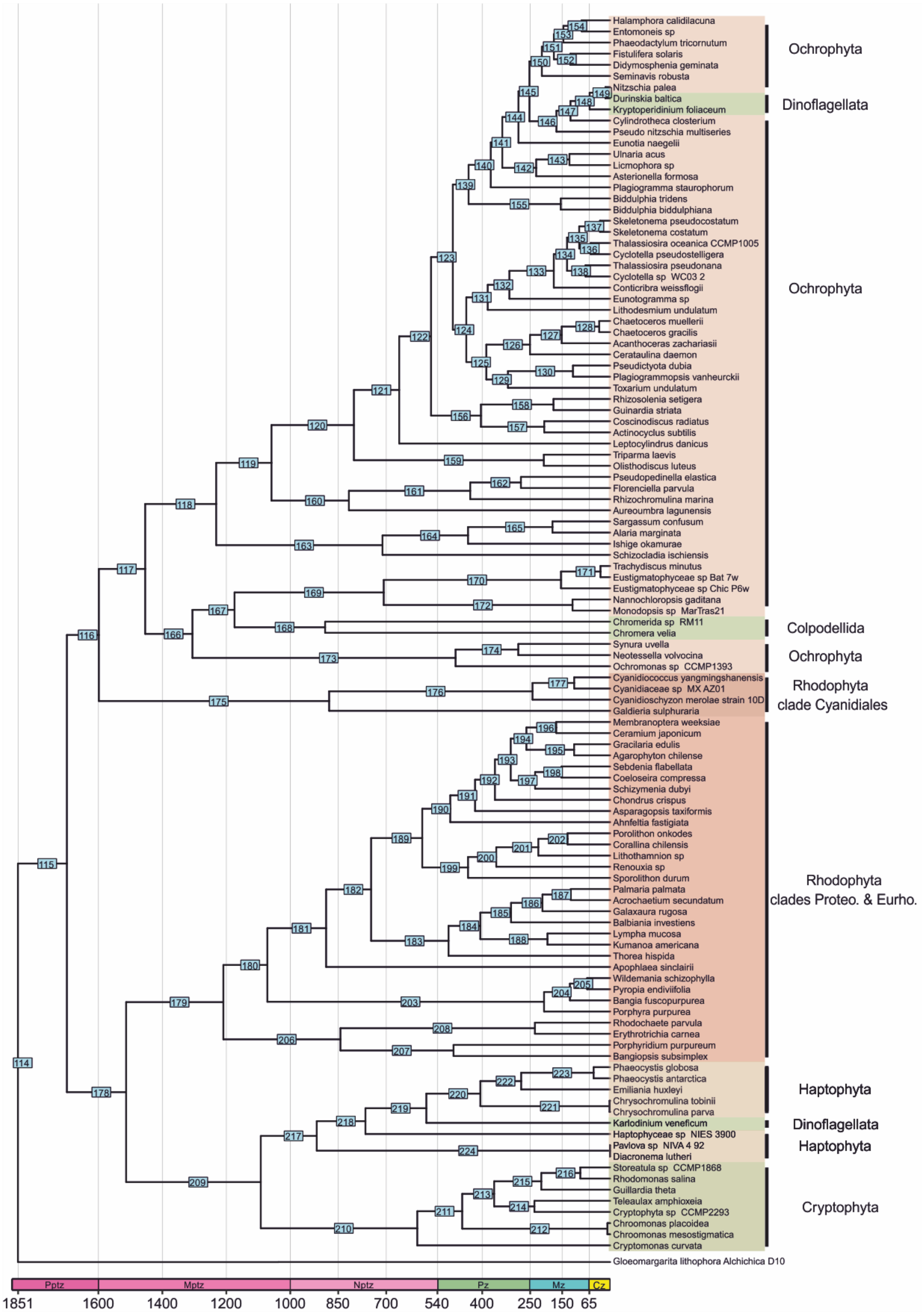

Figure S21. Node numbers to help reading time estimates in Table S4 for the chronogram inferred with Beast using the exponential model (Fig. 2).

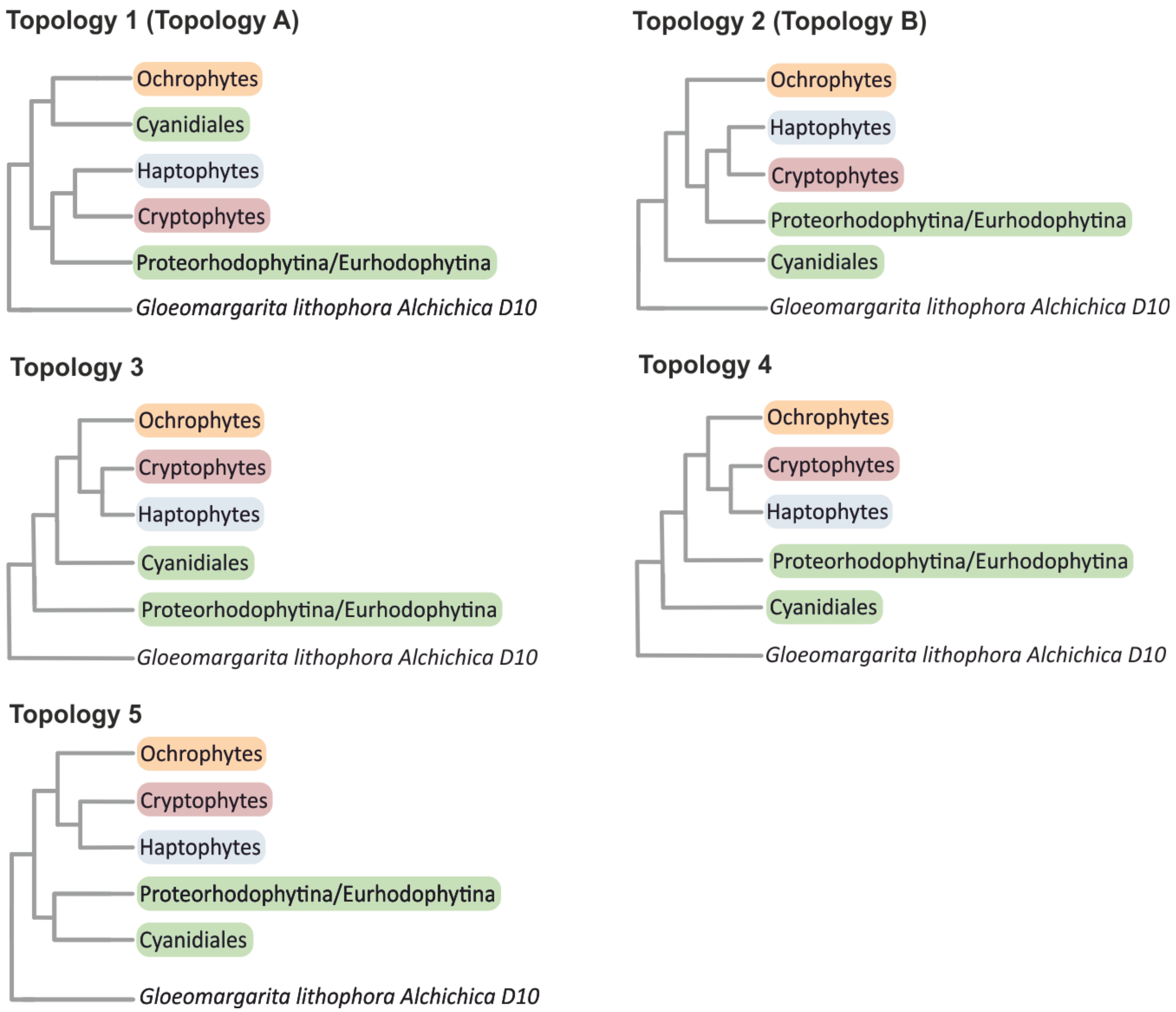

Figure S22. Tree topologies for tests considering the monophyly (topology: 3, 4, and 5) and non-monophyly (topology: 1 - A and 2 - B) of Ochrophytes, Cryptophytes and Haptophytes, as well as the monophyly (topology: 5) and non-monophyly (topology: 1, 2, 3, and 4) of red algae.

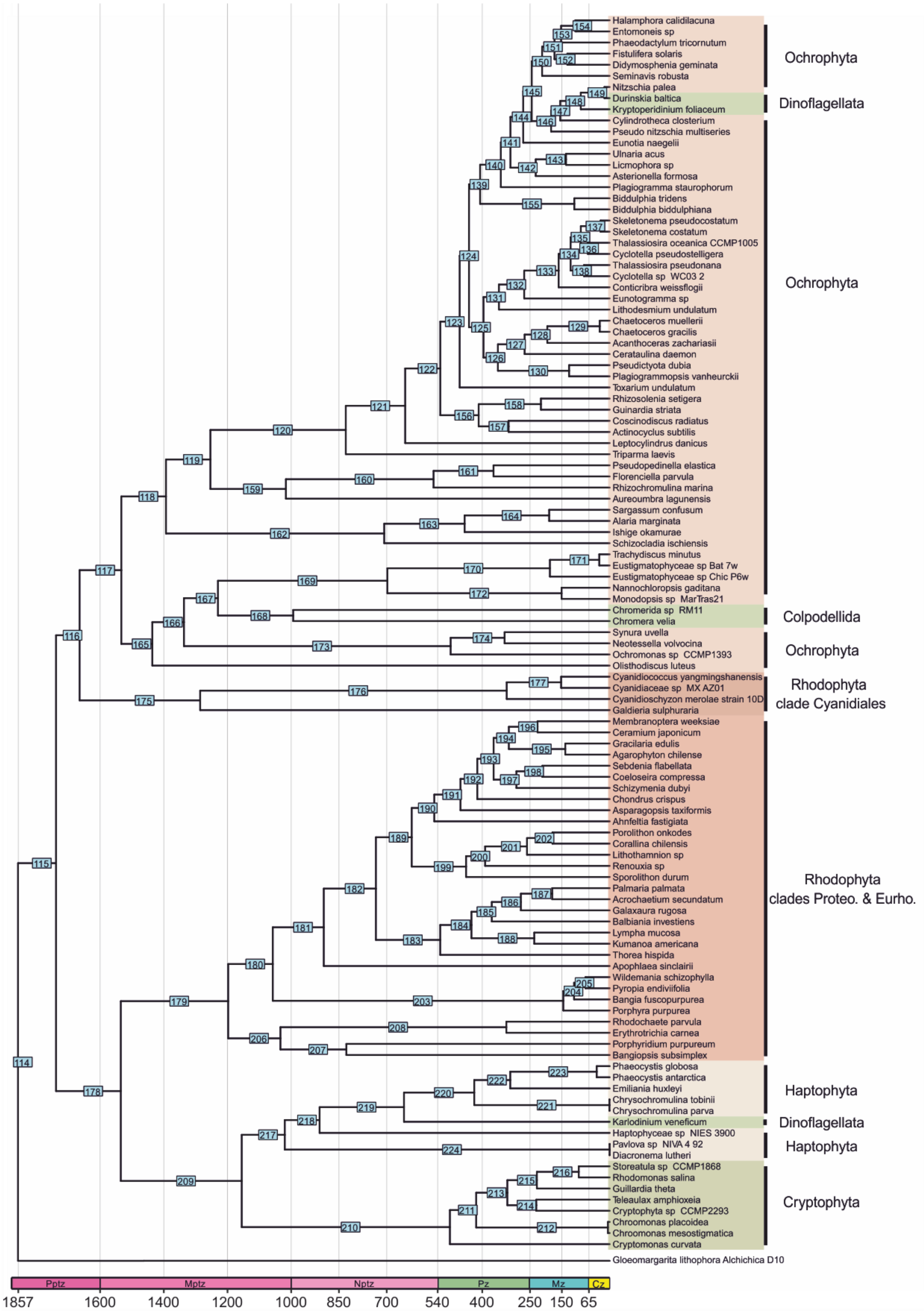

Figure S23. Node numbers to help reading time estimates in Table S9 for the chronogram inferred with Beast using the lognormal model (Fig. S2).

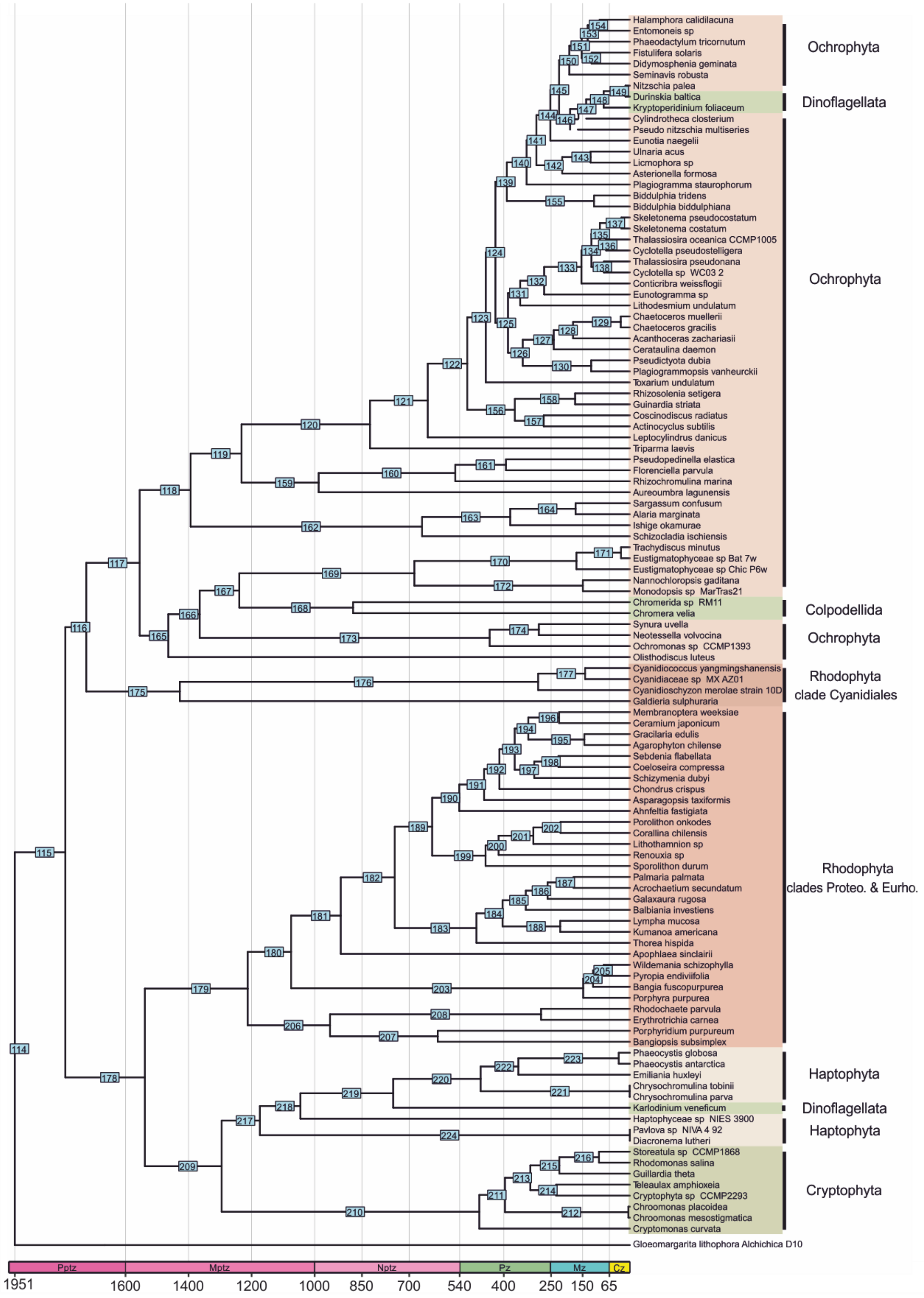

Figure S24. Node numbers to help reading time estimates in Table S10 for the chronogram inferred with MrBayes using the IGR model (Fig. S3).

Table S1. Selected alignment trimming strategies from ClipKIT and trimAl used for maximum likelihood phylogeny inference. The trimming resulted in 49 - 68% sites remaining in the alignments and increased the percent of variable sites from 86% to 95 - 96% and parsimony informative site from 56% to 80 - 89%. The relative composition variability increased very little in the trimmed alignments, from 0.07 to 0.08 - 0.10.

**Trimming strategy**	**Total no. of sites**	**Prc. of left sites**	**No. of variable sites**	**Pct. of variable sites**	**Relative composition variability**	**No. of parsimony informative sites**	**Pct. of parsimony informative sites**
untrimmed alignment	40,342	100%	34,842	86.4%	0.07	22,749	56.4%
**ClipKIT**							
gappy	24,962	62%	23,995	96.1%	0.09	20,979	84.0%
kpic	23,592	58%	22,466	95.2%	0.10	20,894	88.6%
kpic-smart-gap	22,848	57%	21,791	95.4%	0.09	20,361	89.1%
smart-gap	27,506	68%	26,449	96.2%	0.08	22,039	80.1%
**trimAl**							
automated1	21,708	54%	20,742	95.6%	0.08	17,722	81.6%
gappyout	23,102	57%	22,142	95.8%	0.09	19,180	83.0%
strict	19,887	49%	18,923	95.2%	0.08	15,978	80.3%

Table S2. Partitions and substitution models proposed by Partition finder (Lanfear et al., 2017) for Beast (Bouckaert et al., 2014) and ClipKIT smart-gap algorithm (Steenwyk et al., 2020).

Model	Partition no.	Partition range
LG+I+G	Subset 1	1-530
LG+I+G	Subset 2	531-1040
CPREV+G	Subset 3	1041-1251
CPREV+I+G	Subset 4	1252-1418 12449-12514
CPREV+I+G	Subset 5	13012-13387 1419-1614
CPREV+I+G	Subset 6	1615-1787
CPREV+G	Subset 7	11831-11913 1788-1880 11785-11830
CPREV+I+G	Subset 8	22286-22448 1881-2145
LG+G	Subset 9	2146-2498
CPREV+I+G	Subset 10	2499-2961
CPREV+I+G	Subset 11	14815-14917 2962-3303
LG+I+G	Subset 12	3304-3724
LG+I+G	Subset 13	3725-5164
LG+I+G	Subset 14	5165-5829
CPREV+I+G	Subset 15	9618-9659 22891-22980 22578-22707 5830-6370 23809-24043
LG+I+G	Subset 16	6371-6686 26662-27081
CPREV+I+G	Subset 17	27082-27295 6687-7008
LG+I+G	Subset 18	7009-7230
CPREV+G	Subset 19	12082-12147 7231-7401
CPREV+I+G	Subset 20	7402-7439 12189-12361
LG+I+G	Subset 21	7440-7554 9409-9617
CPREV+I+G	Subset 22	7555-7585 12405-12448 24393-24613 24614-24754
JTT+I+G	Subset 23	15393-15440 7586-7627
LG+G	Subset 24	7628-7659 12148-12188
LG+I+G	Subset 25	10839-11342 7660-8414
LG+I+G	Subset 26	12515-13011 8415-9172
LG+G	Subset 27	11955-11994 9173-9258 12041-12081
CPREV+I+G	Subset 28	12362-12404 9259-9408
MTREV+I+G	Subset 29	9660-9705
CPREV+I+G	Subset 30	24755-24896 22168-22285 9888-9919 9706-9887
LG+I+G	Subset 31	9920-10295
LG+I+G	Subset 32	10296-10838
CPREV+I+G	Subset 33	11343-11695
LG+I+G	Subset 34	11696-11784
CPREV+I+G	Subset 35	11914-11954
LG+I+G	Subset 36	11995-12040
LG+I+G	Subset 37	13388-13528
LG+I+G	Subset 38	13529-13650
LG+I+G	Subset 39	13651-13796 23101-23200
CPREV+I+G	Subset 40	23487-23808 14545-14686 13797-13966
CPREV+I+G	Subset 41	13967-14260
CPREV+I+G	Subset 42	15147-15254 14261-14389 22808-22890 23201-23486
LG+I+G	Subset 43	14390-14544
CPREV+I+G	Subset 44	14687-14814
LG+I+G	Subset 45	14918-15146
CPREV+I+G	Subset 46	15255-15343
CPREV+I+G	Subset 47	24897-25896 15344-15392

Model	Partition no.	Partition range
LG+I+G	Subset 48	15441-15676
CPREV+I+G	Subset 49	15677-15894 19249-20109
CPREV+I+G	Subset 50	15895-16347 25897-26359
CPREV+I+G	Subset 51	16348-17560
LG+G	Subset 52	17561-19248
CPREV+I+G	Subset 53	20110-22167
CPREV+I+G	Subset 54	22449-22577
LG+I+G	Subset 55	22708-22807 24044-24280
CPREV+G	Subset 56	22981-23100
LG+I+G	Subset 57	24281-24392
CPREV+I+G	Subset 58	27296-27506 26360-26661

Table S3. Calibration constraints for dating evolution of red alga-derived plastids. We always applied the minimum or maximum constraints on nodes by selecting the lower or upper interval for microfossil dating, respectively. The dates with asterisk represent calculation from previous phylogenetic analyses: 1807 Mya – the minimum age of the common ancestor of Archaeplastida; 2207 Mya – the largest mean estimation from six clocks for the separation of Archaeplastida and *Gloeomargarita*; 573 Mya – the minimum age for the separation of Cryptophyta and Haptophyta; 1701 Mya - the largest mean estimation from six clocks for the separation of Cryptophyta and Haptophyta; the rest constraints represent fossils associated with an appropriate lineage. Importantly, *Rafatazmia chitrakootensis* (node 6) represents controversial fossil, and its inclusion might skew the clocks to provide older dates.

**Node no.**	**Name**	**Age**	**Type**	**References**
1	Gloeomargarita + Archaeplastida	1807 Mya*	MIN	(Strassert et al., 2021)
1,4,5,6	Gloeomargarita + Archaeplastida	2207 Mya*	MAX	(Pietluch et al., 2022)
2	Cryptophyta – Haptophyta	573 Mya*	MIN	(Strassert et al., 2021)
2,3	Cryptophyta – Haptophyta	1701 Mya*	MAX	(Pietluch et al., 2022)
3	The oldest coccolithophores	225 Mya	MIN	(Bown et al., 2004)
4	*Bangiomorpha pubescens*	1030 Mya	MIN	(Gibson et al., 2018)
5	Floridophyceae	595 Mya	MIN	(Xiao et al., 2004)
6	*Rafatazmia chitrakootensis*	1560 Mya	MIN	(Bengtson et al., 2017)
7	Oldest diatoms	185 Mya	MIN	(Kooistra & Medlin, 1996)
7,8,9,10,11	*Rafatazmia chitrakootensis*	1560 Mya	MAX	(Bengtson et al., 2017)
8	diatoms –Coscinodiscophyceae	110 Mya	MIN	(Medlin, 2015)
9	diatoms – Mediophyceae	110 Mya	MIN	(Medlin, 2015)
10	diatoms – *Cyclotella sp.*	24 Mya	MIN	(Kaczmarska et al., 2017)
11	diatoms - earliest pennates	75 Mya	MIN	(Medlin, 2015)

Table S4. Beast molecular clock exponential estimates for nodes presented in Figure S21.

Node	Age mean	Age median	Age 0.95 HPD	Node	Age mean	Age median	Age 0.95 HPD
114	1873,68	1851,61	1800-2018,37	155	168,22	154,48	33,55-350,22
115	1706,76	1698,49	1560-1869,05	156	385,71	404,07	145,41-571,48
116	1598,30	1598,56	1360,23-1819,05	157	234,28	206,22	62,52-464,94
117	1451,45	1453,73	1181,23-1694,96	158	192,12	177,16	36,87-381,26
118	1219,52	1231,83	859,94-1540,72	159	209,10	207,33	169,68-252,72
119	1062,38	1058,91	737,79-1396,98	160	821,14	816,73	455,66-1182,58
120	818,25	801,10	577,04-1084,64	161	443,35	437,58	200,92-688,15
121	663,89	659,62	495,97-843,41	162	295,30	279,00	105,78-520,88
122	563,75	559,89	431,28-699,13	163	717,72	711,65	312,06-1144,54
123	498,87	491,83	387,71-624,13	164	452,61	444,36	124,6-764,61
124	450,42	449,74	325,04-571,82	165	196,06	180,44	53,8-374,77
125	383,19	387,15	238,11-521,63	166	1305,03	1305,95	1043,35-1554,98
126	247,34	250,74	103,99-383,41	167	1179,33	1174,29	937,22-1427,63
127	165,66	151,44	57,59-300,42	168	896,02	891,24	719,15-1079,79
128	41,65	34,19	7,82-98,11	169	694,77	707,92	362,6-1005,43
129	317,40	319,52	153,14-477,31	170	168,50	153,74	40,1-339,95
130	133,19	116,23	36,31-271,47	171	42,28	30,27	5,56-113,63
131	377,81	382,71	213,1-528,27	172	150,48	117,68	28,09-361,81
132	318,26	315,01	149,35-470,22	173	504,88	483,30	186,16-856,64
133	185,32	176,20	89,24-294,57	174	312,87	287,22	82,79-615,28
134	141,89	134,71	72,39-230,57	175	927,91	878,53	398,48-1494,6
135	104,01	96,76	47,94-183,53	176	281,53	242,77	81,3-573,91
136	65,66	60,89	24,69-117,42	177	125,13	112,51	26,34-262,1
137	37,23	31,88	5,3-83,87	178	1498,12	1513,17	1181,64-1757,98
138	82,22	77,09	20,81-153,92	179	1233,92	1209,25	1047,63-1481,62
139	443,95	442,63	335,63-565,5	180	1092,78	1071,65	1020-1233,78
140	375,80	373,20	260,77-481,86	181	876,55	888,01	622,28-1073,61
141	337,79	337,28	241-435,02	182	751,75	747,26	538,13-973,74
142	231,52	231,33	102,75-354,02	183	523,58	505,34	269,38-794,87
143	133,22	127,23	36,83-248,59	184	409,28	405,90	180,76-612,38
144	286,94	286,61	192,3-378,22	185	313,99	309,86	143,63-491,09
145	252,91	252,35	162,26-338,95	186	219,92	211,55	99,15-360,72
146	171,34	167,98	90,45-257,98	187	133,56	123,02	45,8-238,96
147	130,44	124,05	66,75-203,02	188	205,94	196,02	64,19-356,39
148	70,32	64,11	20,52-135	189	605,00	587,45	434,71-819,99
149	18,44	14,83	3,1-46,65	190	506,76	499,96	340,15-691,63
150	215,17	213,54	135,52-294,64	191	436,41	422,33	283,8-633,91
151	177,16	176,00	101,82-246,78	192	378,35	360,37	233,76-561,73
152	127,11	124,55	44,61-213,02	193	322,66	310,90	201,79-473,26
153	147,49	146,02	76,18-228,44	194	273,54	261,82	174,2-439,34
154	94,72	88,88	29,52-166,65	195	116,79	112,77	45,78-194,84

Node	Age mean	Age median	Age 0.95 HPD
196	176,41	168,61	76,99-281,69
197	237,83	234,65	109,35-394,33
198	160,04	152,00	56,24-271,3
199	445,27	444,36	261,31-619,56
200	362,42	356,10	184,86-541,08
201	234,92	224,68	113,95-383,34
202	145,40	133,12	58,81-255,03
203	244,26	206,66	64,58-541,49
204	146,40	125,79	46,72-293,59
205	80,53	72,03	28,94-154,73
206	840,67	843,23	331,63-1332,44
207	519,03	489,56	163,06-954,29
208	276,87	235,17	59,17-602,76
209	1100,67	1092,29	760,94-1456,17
210	625,37	603,06	315,38-939,13
211	494,90	462,54	240,44-807,67
212	18,55	8,10	0,8-72,68
213	389,24	362,80	164,89-668,8
214	261,59	236,79	76,02-520,04
215	251,72	215,37	73,62-525,64
216	114,66	93,36	20,96-277,86
217	932,45	917,25	631,19-1268,09
218	798,27	765,10	542,18-1111,49
219	597,78	574,63	399,35-852,87
220	410,73	405,74	253,72-579,98
221	4,45	1,32	0,09-15,51
222	300,33	278,17	215-449,96
223	62,89	51,73	8,1-146,87
224	1,39	0,32	0,01-4,71

Table S5. Results of tree topology tests considering the monophyly (topology: 3, 4, and 5) and non-monophyly (topology: 1 - A and 2 - B) of Stramenopiles, Cryptophytes and Haptophytes, as well as the monophyly (topology: 5) and non-monophyly (topology: 1, 2, 3, and 4) of red algae. The topologies were shown in Fig. S24. Specifically, we evaluated the support for each topology using the following statistical procedures and tests in IQ-Tree: (i) deltaL: logL difference from the maximal logL in the set; (ii) bp-RELL: bootstrap proportion using RELL method; (iii) p-KH: p-value of one-sided Kishino-Hasegawa test; (iv) p-SH: p-value of Shimodaira-Hasegawa test; (v) p-WKH: p-value of weighted KH test; (vi) p-WSH: p-value of weighted SH test; (vii) c-ELW: Expected Likelihood Weight; (viii) p-AU: p-value of approximately unbiased (AU) test. These analyses were based on the smart-gap trimming alignment, offering supermatrix with the highest number of variable and parsimony informative sites. The topology B was not significantly different from the best topology A but it has a worse log-likelihood value. However, the topologies assuming the monophyly of Ochrophytes, Cryptophytes, and Haptophytes were rejected by all seven or three tests. The p-value from AU test was close to the threshold 0.05 for the topology 4. The p-values smaller than 0.05 were bolded.

**Topology**	**logL**	**deltaL**	**bp-RELL**	**p-KH**	**p-SH**	**p-WKH**	**p-WSH**	**c-ELW**	**p-AU**
1 (A)	-1551912	0	0.533	0.54	1	0.54	0.762	0.533	0.58
2 (B)	-1551914	2.4018	0.433	0.46	0.757	0.46	0.75	0.434	0.557
3	-1552049	137.74	**0**	**0.000231**	**0.000357**	**0.000019**	**0.000049**	**3.36E-19**	**6.9E-109**
4	-1551966	54.367	**0.0326**	0.0923	0.108	**0.0482**	0.128	**0.0327**	0.0561
5	-1552011	99.86	**0.00068**	**0.00772**	**0.0119**	**0.00772**	**0.0231**	**0.000688**	**0.00262**

Table S6. The number of sites with log-likelihood value for the best topology A (topology 1) that are larger/smaller/equal in comparison to the other tree topologies. The topologies were shown in Fig. S24. The number of sites favouring the topology A was larger than for other topologies. The prevalence was more striking when the topologies assuming the monophyly of the studied chromalveolates were compared to the topology A. These analyses were based on the smart-gap trimming alignment, offering supermatrix with the highest number of variable and parsimony informative sites.

Topology	The number of sites		
	**larger**	**smaller**	**equal**
2 (B)	13592	13485	429
3	15554	11759	193
4	14440	12808	258
5	14402	12456	648

Table S7. Results of three tests comparing the number of sites favouring the topology A (topology 1) in comparison to other tree topologies. The excess of these sites was statistically significant according to three or two tests when the best topology was compared with the topologies assuming the monophyly of Stramenopiles, Cryptophytes and Haptophytes. Interestingly, one test also showed that the predominance of these sites is substantially larger for the topology A (topology 1) than for the alternative topology B (topology 2). The p-values smaller than 0.05 were bolded. The topologies were shown in Fig. S24. These analyses were based on the smart-gap trimming alignment, offering supermatrix with the highest number of variable and parsimony informative sites.

**Topology**	**Sign**	**Wilcoxon**	**Proportion**
2 (B)	0.51946	**1.29E-06**	0.366005
3	**1.26E-116**	**1.02E-09**	**1.3E-229**
4	**5.049E-23**	**1.69E-13**	**5.64E-44**
5	**1.733E-32**	0.411437	**8.26E-62**

Table S8. Characteristic of partitions used in the study in terms of supporting the five tree topologies in Fig. S24. The log-likelihood values highest for a given topology were bolded. The number of partitions with the highest log-likelihood values was six or seven for the two topologies, A and B, presenting the non-monophyly of studied chromalveolates, respectively. Two topologies assuming the monophyly of these taxa were each preferred by only two partitions. The topology 3, assuming the monophyly of the chromalveolates and the monophyly of two red algal groups, was supported by no partition. In total, the separation of chromalveolate clades was favoured by 13 out of 17 partitions. What is more, the number of sites present in the partitions supporting the best topology A (topology 1) was the largest, i.e. 17,681 (64% of all 27,506 sites) in comparison to topologies B (topology 2), i.e. 6,636 sites (24%), topology 3 i.e. 1,198 sites (4%) and topology 4 i.e. 1,991 sites (7%). Among the partitions favouring the topology A (topology 1), there is also the largest number of informative sites, i.e. 12,547, whereas in partitions supporting the other topologies are 3,333, 703 and 951 sites, respectively. It indicates that the most important sites are in the partitions associated with topology A produced by more combinations of trimming alignments and methods. These analyses were based on the smart-gap trimming alignment, offering supermatrix with the highest number of variable and parsimony informative sites.

**Partition**	**Alignment sites**			**Log-likelihood values for the given tree topology**				
	**All**	**Infor**	**Invar**	**1 (A)**	**2 (B)**	**3**	**4**	**5**
atpA, psaD, psaJ, psbN	792	464	206	-34113.5	**-34106.4**	-34121.6	-34114.6	-34119.3
atpB	510	229	120	-19188.5	-19189.4	**-19185.4**	-19188.4	-19185.8
atpD, petL, rbcR, rpl19, rpl21, rpl22, rpl23, rpl27, rpl31, rpl34, rps16, rps17, rps18, rps3, rps4, rps8	2464	1912	185	**-178293**	-178301	-178304	-178305	-178300
atpE, atpF, atpG, ycf4	747	659	24	**-63497.6**	-63499.5	-63510.7	-63514	-63514.3
atpH, petB, psaA, psbC	1574	696	608	-40625.8	-40614.4	-40592.9	**-40579.4**	-40585.1
atpI, chlI, petG, psaM, psbF, psbH, psbI, psbZ, rpl14, rpl2, rpl5, rpoC, rps10, rps11, rps13, rps19, rps5, rps7, rps9, ycf3	4657	2090	1681	**-182680**	-182692	-182721	-182711	-182713
cbbX, rbcS, rpl16, rpl36	688	474	86	-34312.6	-34312	**-34309**	-34311.2	-34309.8
ccs1, rpl6, rpoA, rpoC2, rps6, secA	4304	3379	249	**-383992**	-384014	-384022	-384020	-384017
ccsA, groEL, petA, psaF, psaL, psbV, secY, tatC	2534	2130	206	**-195453**	-195463	-195477	-195473	-195473
clpC, rpl33	1529	852	172	-63358.2	**-63357.1**	-63364	-63361.4	-63361.6
dnaK, psaI	707	476	159	-41918.4	**-41914.5**	-41931.6	-41925.9	-41931.2
lysR, rps12, tufA	865	403	327	-25341.3	**-25336.7**	-25343.5	-25337.2	-25345.2
petD, petN, psbJ, psbK, psbT, psbX, psbY	417	255	61	-17947.6	-17941.3	-17945.7	**-17940.7**	-17943.8
petJ, petM, rpl20, rpl3, rpoB, rpoC1, rps14, rps2	2975	2377	227	**-202002**	-202012	-202017	-202013	-202012
psaB, rbcL	1255	606	439	-38876.2	**-38870.3**	-38888.6	-38875.3	-38887.5
psaC, psbB, psbD, psbL	1023	380	322	-21853.7	**-21837.5**	-21857.4	-21842.9	-21856.8
psbA, psbE	465	152	242	-8390.74	**-8385.73**	-8390.31	-8385.85	-8390.5

All – total number of sites

Infor – the number of informative sites

Invar – the number of invariant sites

Table S9. Beast molecular clock lognormal estimates for nodes presented in Figure S22.

Node	Age mean	Age median	Age 0.95 HPD	Node	Age mean	Age median	Age 0.95 HPD
114	1882,17	1857,73	1800-2045,4	155	120,98	110,99	38,83-225,34
115	1741,20	1738,31	1560-1930,14	156	407,66	411,38	237,33-552,59
116	1670,17	1664,49	1462,88-1888,32	157	319,64	317,26	156,35-465,44
117	1538,84	1533,53	1337,32-1743,7	158	229,47	215,79	91,89-409,88
118	1397,18	1393,25	1153,87-1642,45	159	1008,58	1016,59	644,92-1355,32
119	1257,57	1253,75	992,09-1527,41	160	556,76	552,90	302,54-805,68
120	836,13	828,59	597,44-1101,75	161	378,05	364,66	171,02-618,94
121	651,02	642,01	472,95-830,45	162	726,69	707,88	462,94-1065,08
122	536,88	531,77	425-665,58	163	469,64	455,49	236,55-728,38
123	473,50	471,01	383,27-571,18	164	206,94	189,92	61,61-377,44
124	442,90	441,70	358,56-527,96	165	1439,55	1436,09	1229,7-1651,16
125	396,50	396,06	313,54-480,02	166	1342,24	1336,80	1123,51-1564,38
126	349,84	350,82	251,23-441,46	167	1233,43	1229,92	1023,47-1454,28
127	266,74	266,94	158,04-378,99	168	996,78	993,68	787,85-1204,65
128	200,96	195,82	105,15-306,1	169	698,43	697,91	426,67-993,89
129	33,55	31,11	8,44-62,59	170	195,78	187,58	67,49-326,78
130	136,12	127,38	31,47-251,66	171	36,74	32,54	7,23-72,62
131	346,89	347,73	253,02-428,02	172	168,64	150,99	57,67-331,38
132	268,88	268,41	183,64-354,76	173	498,55	499,31	183,4-753,62
133	162,48	160,24	95,35-226,05	174	337,19	329,86	100,55-572,36
134	124,77	122,67	72,43-175,93	175	1238,62	1285,80	679,82-1648,02
135	93,03	90,50	49,4-138,43	176	349,83	323,24	109,65-630,95
136	71,30	68,50	33,28-117,38	177	172,93	152,36	47,55-350,05
137	32,17	28,58	7,31-66,62	178	1538,97	1535,04	1272,84-1802,51
138	83,66	80,65	34,16-139,13	179	1210,25	1197,89	1059,01-1386,9
139	407,17	406,77	325,42-484,73	180	1074,50	1057,89	1020-1183,44
140	341,36	341,66	269,65-408,48	181	889,53	897,72	673,32-1075,46
141	310,88	311,52	243,68-379,05	182	736,79	734,02	551,93-918,02
142	234,12	232,11	157,45-319,53	183	533,65	532,02	339,25-717,88
143	139,45	137,03	66,92-221,12	184	437,62	434,01	263,63-599,44
144	271,33	271,33	210,24-327,95	185	373,39	370,06	210,84-537,52
145	245,09	244,71	190,58-297,87	186	281,94	278,82	139,03-425,03
146	185,08	183,96	135,21-237,56	187	182,70	180,39	53,01-307,51
147	156,58	155,27	108,43-209,69	188	248,81	236,73	103,05-416,23
148	92,69	90,63	43,56-142,23	189	623,48	621,54	468,11-785,42
149	18,31	16,35	4,53-36,87	190	551,33	550,66	392,74-716,85
150	213,16	212,16	160,45-267,49	191	471,20	468,39	340,09-624,71
151	174,27	173,24	120,6-227,25	192	420,26	415,69	300-571,45
152	133,53	132,88	70,05-204,6	193	371,29	364,44	248,72-511,71
153	154,05	152,45	100,83-211,75	194	322,11	316,53	198,24-456,18
154	111,56	108,14	57,16-174,21	195	143,78	139,41	68,79-221,87

Node	Age mean	Age median	Age 0.95 HPD
196	234,51	226,42	120,57-356,05
197	294,29	292,75	163,72-440,27
198	212,26	211,33	96,81-329,19
199	455,54	450,90	303,21-620,87
200	394,71	391,01	245,58-542,35
201	265,81	259,44	143,89-398,79
202	187,58	179,62	91,75-295,36
203	155,64	145,57	69,63-272,79
204	122,50	111,75	44,21-212,7
205	84,31	75,80	27,09-162,78
206	1022,00	1033,86	725,57-1283,6
207	801,69	826,73	406,22-1181,4
208	361,50	324,55	83,57-729,42
209	1156,68	1155,27	797,67-1486,71
210	512,84	501,60	314,69-724,55
211	431,44	419,08	264,34-629,19
212	6,19	5,44	1,37-12,52
213	331,73	321,28	193,97-488,24
214	242,01	230,38	125,77-386,36
215	239,87	228,57	125,68-380,34
216	101,76	97,12	31,33-177,76
217	1020,21	1020,22	701,85-1322,96
218	912,41	910,78	600,7-1188,66
219	655,78	645,80	441,5-881,2
220	437,27	425,25	275,18-622,35
221	0,84	0,71	0,12-1,96
222	327,85	312,18	215-485,43
223	46,36	40,64	10,45-94,72
224	0,23	0,18	0,01-0,58

Table S10. MrBayes molecular clock IGR estimates for nodes presented in Figure S23.

Node	Age mean	Age median	Age 0.95 HPD	Node	Age mean	Age median	Age 0.95 HPD
114	1997,67	1951,24	1807-2328,73	155	125,39	113,99	51,19-228,69
115	1806,96	1790,95	1560,01-2075,65	156	372,88	364,93	159,51-578,9
116	1739,97	1724,53	1493,79-2016,16	157	283,51	273,29	86,68-481,26
117	1567,70	1554,96	1319,81-1848,73	158	196,42	173,78	58,5-372
118	1389,45	1393,35	1029,51-1710,08	159	994,94	987,30	605,33-1357,28
119	1237,29	1231,95	916,04-1537,71	160	558,70	553,92	264,25-869,76
120	854,61	824,56	592,24-1150,57	161	394,80	393,16	185,08-630,58
121	651,57	641,18	434,26-862,06	162	676,19	659,20	351,5-1027,97
122	521,43	516,04	365,59-675,19	163	391,08	379,70	198,01-582,96
123	462,32	457,20	313,68-599,69	164	182,60	172,28	81,41-296,82
124	431,37	426,62	303,02-570,39	165	1475,11	1464,20	1216,98-1762,46
125	388,22	386,70	251,14-514,82	166	1371,66	1364,66	1120,3-1639,49
126	341,81	339,83	206,12-473,45	167	1245,80	1239,11	1007,58-1503,34
127	246,56	241,66	140,29-379,3	168	885,78	878,28	671,43-1111,13
128	185,34	180,27	91,48-298,76	169	681,74	683,71	412,47-926,76
129	33,77	28,92	9,74-68,7	170	181,23	169,67	69,95-315,55
130	131,06	122,91	50,91-227,35	171	34,49	28,11	9,32-77,46
131	348,69	348,08	228,02-471,92	172	157,31	149,28	39,84-278,79
132	275,85	272,02	182,25-373,67	173	458,31	444,79	228-707,84
133	158,88	153,92	95,99-226,53	174	307,59	288,87	127,62-514,93
134	124,76	122,08	76,31-176,38	175	1407,46	1427,76	860,96-1854,62
135	97,39	95,24	54,19-139,42	176	308,48	291,21	131,72-505,67
136	77,20	75,36	39,55-114,65	177	156,76	142,17	65,66-284,83
137	29,62	26,91	10,22-54,84	178	1543,38	1538,51	1210,42-1882,39
138	84,87	82,34	39,34-138,55	179	1231,50	1212,41	1077,06-1434,65
139	395,68	390,33	273,97-532,98	180	1094,10	1074,87	1030-1223,66
140	328,93	327,62	225,14-430,04	181	907,86	917,14	653,13-1132,57
141	297,67	296,66	193-382,47	182	746,74	745,89	551,12-955,89
142	219,61	214,82	141,9-312,39	183	487,84	486,86	293,57-674,33
143	132,09	124,85	62,73-217,02	184	409,39	404,00	239,77-554,34
144	252,60	252,35	170,07-327,86	185	338,08	330,18	206,91-481,45
145	224,89	224,57	148,86-288,87	186	268,18	261,18	161,54-409,94
146	168,90	165,15	108,78-232,04	187	188,39	178,38	90,45-301,27
147	146,19	138,74	89,6-210,43	188	237,05	221,24	118,73-406,89
148	87,81	83,18	38,04-143,11	189	629,64	627,28	466,66-795,22
149	15,56	14,02	5,08-30,43	190	550,84	541,35	395,58-736,29
150	193,12	192,23	129,84-253,69	191	468,27	462,57	319,46-625,39
151	154,87	152,96	100,71-209,15	192	417,18	413,94	289,01-562,82
152	123,77	121,54	74,48-180,76	193	369,19	365,40	261,91-498,54
153	136,23	133,94	88,31-189,98	194	323,07	322,30	218,31-441,1
154	97,76	95,94	52,73-145,28	195	147,54	144,58	74,74-229,17

Node	Age mean	Age median	Age 0.95 HPD
196	226,24	224,56	124,54-325,7
197	304,07	303,55	179-425,39
198	232,64	226,71	132,04-361,54
199	468,74	457,91	316,35-642,04
200	420,83	416,46	262,92-586,71
201	306,60	306,29	191,99-426,62
202	221,27	220,17	122,02-322,42
203	164,34	148,19	80,71-296,86
204	129,09	116,88	59,1-242,46
205	92,96	83,01	39,7-175,92
206	903,30	951,28	439,82-1259,88
207	652,56	609,58	284,42-1099,17
208	327,48	281,91	104,44-708,23
209	1290,46	1294,99	848,99-1722,3
210	505,81	477,77	293,72-770,76
211	420,88	396,35	237,84-665,78
212	5,58	4,78	1,5-11,76
213	334,15	315,84	205,11-522,27
214	248,09	234,04	127,81-392,75
215	241,56	223,92	133,22-403,31
216	102,41	98,09	38,19-171,2
217	1170,92	1173,71	741,09-1554,75
218	1049,97	1045,26	665,89-1401,65
219	754,47	750,95	472,28-1057,59
220	495,29	473,50	307,42-729,24
221	0,82	0,68	0,16-1,84
222	372,18	353,89	225-548,48
223	42,06	35,37	10,51-88,13
224	0,23	0,16	0-0,6

Table S11. Comparison of molecular clock estimates from various studies for the divergence of the crown groups of Stramenopiles, plastid-containing Alveolata, Cryptista and Haptista.

	Strassert et al. 2021		Parfrey et al. 2011	Berney at al. 2006	Yoon at al. 2004	Douzery et al. 2004
**Crown Group**	**MIN**	**MAX**	**MEAN**	**MEAN**	**MEAN**	**MEAN**
Stramenopiles	818,6	1224,33	760	575	765	
Plastid containing Alveolata	860,17	1402,5	1100	475	-	600
Cryptista	529,78	747,57	800	-	860	
Haptista	931,09	1295,87	680	560	787	
**Markers**	320 nuclear-encoded proteins		15 nuclear-encoded protein genes	plastid-encoded SSU rRNA gene	plastid-encoded SSU rRNA, *psaA*, *psaB*, *psbA*, *rbcL*, and *tufA* genes and PsaA, PsaB, PsbA, RbcL, and TufA proteins	129 nuclear-encoded proteins

Table S12. Number of missing markers for investigated species. We wanted to investigate the greatest number of species carrying red-alga derived plastids sharing the greatest number of markers. The selected data set was a compromise between the number of taxa and markers. Only three species lacked a substantial number of markers from the data set; however, the elimination of these species did not affect the phylogenetic results (data not shown). Matrix visualizing the species and their markers is accessible at the zenodo repository, see the section Data and Resource Availability.

**Number of missing markers from the dataset**	**Number of species**
2	23
3	63
4	11
5	5
6	5
7	1 (*Ochromonas* sp. CCMP1393)
10	1 (*Aureoumbra lagunensis*)
36	1 (*Chromerida* sp. RM11)
47	1 (*Karlodinium veneficum*)
48	1 (*Chromera velia*)

Table S13. Partitions and substitution models proposed by Partition finder (Lanfear et al., 2017) for MrBayes (Ronquist et al., 2012) and ClipKIT smart-gap algorithm (Steenwyk et al., 2020).

Model	Partition no.	Partition range
WAG+I+G	Subset1	1-530
CPREV+G	Subset2	531-1040
CPREV+G	Subset3	1041-1251
CPREV+I+G	Subset4	12449-12514 1252-1418
CPREV+I+G	Subset5	13012-13387 1419-1614
CPREV+G	Subset6	1615-1787
CPREV+I+G	Subset7	11831-11913 1788-1880 11785-11830 15393-15440
CPREV+I+G	Subset8	1881-2145 23101-23200 2146-2498 22286-22448
CPREV+I+G	Subset9	24281-24392 2499-2961
CPREV+G	Subset10	2962-3303
CPREV+I+G	Subset11	3304-3724
VT+I+G	Subset12	3725-5164
CPREV+I+G	Subset13	5165-5829
CPREV+I+G	Subset14	9618-9659 22578-22707 22891-22980 23809-24043 5830-6370
CPREV+I+G	Subset15	6371-6686
CPREV+I+G	Subset16	27082-27295 6687-7008
CPREV+G	Subset17	7009-7230
CPREV+I+G	Subset18	7231-7401 12082-12147
CPREV+I+G	Subset19	7402-7439 12189-12361
CPREV+I+G	Subset20	7440-7554 14815-14917
CPREV+I+G	Subset21	7555-7585 12405-12448 24393-24613 15441-15676 24614-24754
CPREV+G	Subset22	7586-7627 9888-9919 22708-22807
MTREV+I+G	Subset23	7628-7659
CPREV+I+G	Subset24	7660-8414 11914-11954 10839-11342
CPREV+I+G	Subset25	8415-9172
CPREV+I+G	Subset26	9173-9258 11343-11695 12041-12081
CPREV+I+G	Subset27	9259-9408 13651-13796 12362-12404
CPREV+I+G	Subset28	9409-9617 15255-15343
MTREV+I+G	Subset29	9660-9705
CPREV+I+G	Subset30	24755-24896 22168-22285 9706-9887
CPREV+I+G	Subset31	9920-10295
CPREV+I+G	Subset32	10296-10838
CPREV+G	Subset33	11696-11784
CPREV+I+G	Subset34	11955-11994 12148-12188
MTMAM+I+G	Subset35	11995-12040
RTREV+I+G	Subset36	12515-13011
WAG+I+G	Subset37	13388-13528
RTREV+I+G	Subset38	13529-13650
CPREV+I+G	Subset39	23487-23808 14545-14686 13797-13966
CPREV+I+G	Subset40	13967-14260
CPREV+I+G	Subset41	14261-14389 22808-22890 14918-15146 15147-15254 24044-24280 23201-23486
CPREV+I+G	Subset42	14390-14544 15344-15392
CPREV+I+G	Subset43	14687-14814
CPREV+I+G	Subset44	19249-20109 15677-15894
CPREV+I+G	Subset45	25897-26359 15895-16347
CPREV+I+G	Subset46	16348-17560
VT+I+G	Subset47	17561-19248

Model	Partition no.	Partition range
CPREV+I+G	Subset48	20110-22167
CPREV+G	Subset49	22449-22577
CPREV+I+G	Subset50	22981-23100
CPREV+I+G	Subset51	24897-25896
CPREV+I+G	Subset52	27296-27506 26360-26661
WAG+I+G	Subset53	26662-27081

Table S14. Partitions and substitution models proposed by ModelFinder (Kalyaanamoorthy et al., 2017) for IQ-TREE (Minh et al., 2020) and ClipKIT gappy algorithm (Steenwyk et al., 2020).

Model	Partition no.	Partition range
LGF	Subset 1	1-505 8336-8477 8706-8748 10999-11041
LG	Subset 2	506-981
LGF	Subset 3	982-1168 11869-12186 12587-12712 13108-13223 13224-13345 13346-13449 13450-13536 13537-13745 13746-13821 13984-14221 17918-18669 20931-21017 21188-21429 21430-21653 21654-21859 22311-22442
JTTF	Subset 4	1169-1305 1306-1491 1492-1654 24773-24962
mtZOAF	Subset 5	1655-1737 6357-6516 6739-6768 6769-7520 10839-10876 10877-10915 11042-11073
mtInvF	Subset 6	1738-1987 6666-6696 11238-11277 11315-11378
LG	Subset 7	1988-2291 12448-12586
LGF	Subset 8	2292-2736 14222-14402 14403-14725 18670-20256 22047-22154 22581-23483
mtInvF	Subset 9	2737-3061 5822-6141 8478-8668 11074-11237 23484-23915 23916-24178
LGF	Subset 10	3062-3415 4972-5504 6517-6553 6697-6738 8749-8906 8907-8936 11278-11314 12187-12325 12713-12988 12989-13107 13936-13983 16220-17917 20257-20365 20366-20495 20622-20748 21095-21187 21860-22046 22155-22310 22443-22580 24588-24772
LG	Subset 11	3416-4344 6554-6665
LGF	Subset 12	4345-4971 8669-8705
LG	Subset 13	5505-5821 10916-10960 20496-20621 24179-24587
LGF	Subset 14	6142-6356 8255-8335 8937-9296 9297-9805 10293-10643 10644-10727 10961-10998
LG	Subset 15	7521-8254 9806-10292 10728-10771 10772-10838 11379-11868 12326-12447
JTTDCMutF	Subset 16	13822-13887 13888-13935 14726-16219 20749-20848 20849-20930 21018-21094

Table S15. Partitions and substitution models proposed by ModelFinder (Kalyaanamoorthy et al., 2017) for IQ-TREE (Minh et al., 2020) and ClipKIT kpic algorithm (Steenwyk et al., 2020).

**Model**	**Partition no.**	**Partition range**
LGF	Subset 1	1-452 7725-7856 8128-8298 20816-20978
LG	Subset 2	453-814 5343-5615 22858-23211
LGF	Subset 3	815-1047 10818-11145 11495-11632 11996-12127 12128-12262 12263-12375 12376-12468 12469-12667 12668-12755 16484-17297 19569-19653 19654-19750 19839-20072 20073-20318 20319-20520
mtInvF	Subset 4	1048-1191 1192-1378 1379-1540 2537-2864 5616-5911 7857-8043 10076-10228 20521-20703 22140-22589 22590-22857 23392-23592
mtZOAF	Subset 5	1541-1598 5912-6094 6095-6205 6399-6426 6427-7073 7673-7724 8652-9058 9059-9476 9477-9695 9696-9766 9800-9858 9859-9896 9971-9998 10049-10075
mtZOAF	Subset 6	1599-1816 6206-6238 6335-6363 6364-6398 8084-8127 8299-8328 9930-9970 9999-10048 10270-10307 10308-10371 11266-11371 19184-19288
LGF	Subset 7	1817-2099 2865-3188 4812-5342 10229-10269 11146-11265 11372-11494 11633-11886 12871-12913 12914-13138 18940-19058 19059-19183 19289-19402 19751-19838 20979-21104 21105-21239 23212-23391
LGF	Subset 8	2100-2536 12824-12870 13319-13719 19490-19568
LG	Subset 9	3189-4204 6239-6334
LGF	Subset 10	4205-4811 8044-8083
LG	Subset 11	7074-7672 9767-9799 9897-9929 10372-10817
LG	Subset 12	8329-8651
JTTDCMutF	Subset 13	11887-11995 12756-12823 13720-15187 19403-19489
LGF	Subset 14	13139-13318 17298-18939 20704-20815 21240-22139
LG	Subset 15	15188-16483

Table S16. Partitions and substitution models proposed by ModelFinder (Kalyaanamoorthy et al., 2017) for IQ-TREE (Minh et al., 2020) and ClipKIT kpic-smart-gap algorithm (Steenwyk et al., 2020).

Model	Partition no.	Partition range
Q.yeast	Subset 1	1-447 4062-4659 7561-7689 7958-8121
Q.yeast	Subset 2	448-796 5181-5453 18502-18604 22124-22477
Q.plantF	Subset 3	797-1002 10633-10946 11296-11433 11796-11927 11928-12051 12052-12161 12162-12252 12253-12448 12449-12536 13469-14566 15863-16618 18261-18376 18885-18969 18970-19055 19142-19370 19371-19616 19617-19814 20108-20270 20396-20526
Q.yeast	Subset 4	1003-1146 1147-1332 1333-1494 4660-5180 7690-7876 18719-18805 22658-22848
mtZOAF	Subset 5	1495-1550 5749-5931 5932-6042 6235-6262 6263-6909 6910-7508 7509-7560 8152-8474 9300-9515 9516-9586 9620-9678 9717-9749 9750-9790
mtZOAF	Subset 6	1551-1768 6043-6075 6171-6199 6200-6234 7914-7957 8122-8151 9818-9867 10044-10084 10085-10122 10123-10186 11067-11172 18605-18718
Q.yeast	Subset 7	1769-2043 2787-3105 7877-7913 10947-11066 11173-11295 11434-11687 11688-11795 12651-12692 12693-12915 18377-18501 19056-19141 19815-19997 20271-20395 22478-22657
Q.plantF	Subset 8	2044-2477 12605-12650 13096-13468 18806-18884
mtInvF	Subset 9	2478-2786 5454-5748 6076-6170 9892-10043 21416-21857 21858-22123
Q.yeast	Subset 10	3106-4061 12537-12604
Q.yeast	Subset 11	8475-8881 9679-9716 9791-9817 9868-9891
Q.yeast	Subset 12	8882-9299 9587-9619 10187-10632
Q.pfamF	Subset 13	12916-13095 16619-18260 19998-20107 20527-21415
LG	Subset 14	14567-15862

Table S17. Partitions and substitution models proposed by ModelFinder (Kalyaanamoorthy et al., 2017) for IQ-TREE (Minh et al., 2020) and ClipKIT smart-gap algorithm (Steenwyk et al., 2020).

Model	Partition no.	Partition range
Q.yeast	Subset 1	1-530 9259-9408 9660-9705 12082-12147
LG	Subset 2	531-1040
LGF	Subset 3	1041-1251 7555-7585 13012-13387 13797-13966 14390-14544 14545-14686 14687-14814 14815-14917 15147-15254 15344-15392 22808-22890 22891-22980 22981-23100 23487-23808 23809-24043 24614-24754
Q.plantF	Subset 4	1252-1418 1419-1614 1615-1787 27296-27506
mtZOAF	Subset 5	1788-1880 7009-7230 7660-8414 10839-11342
Q.yeast	Subset 6	1881-2145 3304-3724 7402-7439 9888-9919 11785-11830 11831-11913 11914-11954 12449-12514 13529-13650 13967-14260 15441-15676 17561-19248 22168-22285 22286-22448 22578-22707 23101-23200 24044-24280 24393-24613 24755-24896 27082-27295
Q.yeast	Subset 7	2146-2498 13388-13528 13651-13796 15393-15440
Q.plantF	Subset 8	2499-2961 15677-15894 15895-16347 20110-22167 24281-24392 24897-25896
mtInvF	Subset 9	2962-3303 5830-6370 6687-7008 9409-9617 9706-9887 12189-12361 25897-26359 26360-26661
Q.yeast	Subset 10	3725-5164 15255-15343
LGF	Subset 11	5165-5829 9618-9659
Q.yeast	Subset 12	6371-6686 22449-22577 26662-27081
LGF	Subset 13	7231-7401 7628-7659 11955-11994 11995-12040 12148-12188 12362-12404 12405-12448
Q.yeast	Subset 14	7440-7554 7586-7627 14261-14389 14918-15146 16348-17560 19249-20109 22708-22807 23201-23486
Q.yeast	Subset 15	8415-9172 12515-13011
Q.yeast	Subset 16	9173-9258 10296-10838 11343-11695 12041-12081
Q.yeast	Subset 17	9920-10295 11696-11784

Table S18. Partitions and substitution models proposed by ModelFinder (Kalyaanamoorthy et al., 2017) for IQ-TREE (Minh et al., 2020) and trimAl automated1 algorithm (Capella-Gutiérrez et al., 2009).

Model	Partition no.	Partition range
Q.yeast	Subset 1	1-502 1385-1507 3879-4446 6144-6180 7764-7905 12931-12999
Q.yeast	Subset 2	503-965 4969-5283 10282-10325 20974-21381
LGF	Subset 3	966-1129 2096-2454 13000-13062 13063-13101 13361-13509 16770-17714 19504-19633 19746-20463
Q.plantF	Subset 4	1130-1243 1244-1384 21542-21708
Q.yeast	Subset 5	1508-1588 8691-9194 10016-10098 10326-10363
mtZOAF	Subset 6	1589-1813 2455-2715 5284-5603 6122-6143 10437-10599 10673-10724 20464-20765 20766-20973
Q.yeast	Subset 7	1814-2095 3068-3878 8122-8162 10638-10672 11497-11634 11754-11887 16275-16769 17801-17920
Q.yeast	Subset 8	2716-3067 5978-6013 14613-16274
Q.yeast	Subset 9	4447-4968 8163-8304 8305-8333 11212-11496 11888-11972 12247-12358 12359-12448 12449-12562 12563-12646 12647-12724 12725-12930 13149-13360 13510-13764 17715-17800 18044-18164 18264-18319 18320-18391 18392-18451 18541-18759 18927-19126 19265-19348
mtZOAF	Subset 10	5604-5818 6210-6951 7684-7763 8334-8690 9195-9664 9665-10015 10206-10242
LGF	Subset 11	5819-5977 6181-6209 10243-10281 10406-10436 10600-10637
Q.yeast	Subset 12	6014-6121 7906-8089 11973-12246 18165-18263
Q.yeast	Subset 13	6952-7683 10099-10140 10725-11211 17921-18043
Q.yeast	Subset 14	8090-8121 10141-10205 10364-10405 11635-11753 13102-13148 13765-14612 18452-18540 18760-18926 19127-19264 19349-19503 19634-19745 21382-21541

Table S19. Partitions and substitution models proposed by ModelFinder (Kalyaanamoorthy et al., 2017) for IQ-TREE (Minh et al., 2020) and trimAl gappyout algorithm (Capella-Gutiérrez et al., 2009).

Model	Partition no.	Partition range
Q.yeast	Subset 1	1-502 7968-8109 10303-10344 10410-10446
Q.yeast	Subset 2	503-965 5160-5474 22356-22763
LGF	Subset 3	966-1149 1276-1451 1452-1591 4070-4637 6205-6312 6343-6384 8110-8293 13207-13275 19157-19255 22924-23102
mtInvF	Subset 4	1150-1275 2597-2906 5475-5794 6313-6342 10641-10803 21758-22110 22111-22355
mtZOAF	Subset 5	1592-1672 5795-6009 6414-7155 7888-7967 8538-8894 8895-9398 9399-9868 9869-10219 10220-10302 10447-10485 10530-10567 10610-10640
LGF	Subset 6	1673-1897 6010-6168 8326-8366 10804-10842 10878-10935 11720-11857 13383-13429 18793-18912
Q.yeast	Subset 7	1898-2179 3259-4069 6385-6413 10568-10609 10843-10877 11977-12110
LGF	Subset 8	2180-2596 13276-13338 13339-13382 13666-13843 13844-14147 17494-18690 18691-18792 20869-21757
Q.yeast	Subset 9	2907-3258 6169-6204 12220-12493 15206-16867
Q.yeast	Subset 10	4638-5159 8367-8508 8509-8537 12494-12605 12710-12823 13430-13665 14148-15205 16868-17493 19036-19156 19333-19410 19480-19568 19569-19787 20193-20359 20455-20609 20740-20868
Q.yeast	Subset 11	7156-7887 10486-10529 10936-11422 18913-19035
Q.yeast	Subset 12	8294-8325 10345-10409 11858-11976 22764-22923
LGF	Subset 13	11423-11719 12111-12219 12606-12709 12824-12922 12923-13000 13001-13206 19256-19332 19411-19479 19788-19992 19993-20192 20360-20454 20610-20739

Table S20. Partitions and substitution models proposed by ModelFinder (Kalyaanamoorthy et al., 2017) for IQ-TREE (Minh et al., 2020) and trimAl strict algorithm (Capella-Gutiérrez et al., 2009).

Model	Partition no.	Partition range
LGF	Subset 1	1-434 9315-9453
LG	Subset 2	435-843 4470-4759 9187-9223 16325-16432
LGF	Subset 3	844-1007 1890-2248 10023-10307 11249-11332 11986-12134 15200-16144 16539-16625 16754-16813 16889-17086 17560-17643 18001-18718
LGF	Subset 4	1008-1121 1263-1385 3472-3993 5490-5526 7072-7237 11158-11248 11697-11735 17644-17771
mtZOAF	Subset 5	1122-1262 2249-2509 4760-5021 11333-11401 11646-11696 18719-19020 19021-19228 19721-19887
LG	Subset 6	1386-1456 8950-9027 9061-9117 9286-9314
mtZOAF	Subset 7	1457-1649 5468-5489 7238-7261 7295-7419 9150-9186 9454-9491 9522-9573 16231-16324
LG	Subset 8	1650-1889 9252-9285 10426-10529 14705-15199
LG	Subset 9	2510-2793
LG	Subset 10	2794-3471 10530-10648 16814-16888 19583-19720
LGF	Subset 11	3994-4469 5382-5467 7420-7445 9492-9521 10308-10425 10649-10733 10734-10973 10974-11067 11068-11157 11402-11577 11578-11645 11736-11773 11774-11985 12135-12389 12390-13237 16145-16230 16433-16538 16626-16681 16682-16753 17087-17253 17254-17421 17422-17559 17772-17888 17889-18000
mtZOAF	Subset 12	5022-5214 5351-5381 5550-6240 6890-6963 7446-7758 7759-8220 8221-8648 8649-8949 9028-9060 9224-9251
mtZOA	Subset 13	5215-5350 5527-5549
LG	Subset 14	6241-6889 9118-9149 9574-10022
LG	Subset 15	6964-7071 7262-7294 13238-14704 19229-19582

Table S21. Partitions and substitution models proposed by ModelFinder (Kalyaanamoorthy et al., 2017) for IQ-TREE (Minh et al., 2020) and original alignment (no trimming strategy).

**Model**	**Partition no.**	**Partition range**
Q.yeast	Subset 1	1-611 12611-12775
LG	Subset 2	612-1534
Q.yeast	Subset 3	1535-2057 7668-8358 10332-10377 12776-13003 18445-18596 19508-19645
Q.plantF	Subset 4	2058-2242 2243-2460 2461-2647 40061-40342
Q.insect	Subset 5	2648-2837 15490-15539 15540-15644 17483-17698
mtInvF	Subset 6	2838-3143 10300-10331 13004-13048 15882-15978 16241-16292 16293-16337 16338-16414 19797-19848
Q.yeast	Subset 7	3144-3873 4837-5524 10106-10163 13329-13361 17332-17482 17699-17860 18111-18444 23812-25509 29961-30092 30093-30661 30808-30961 31430-31727 31728-31894 35419-36019 36177-36605 37010-37222 39822-40060
Q.plantF	Subset 8	3874-4381 20599-21408 27082-29960 31119-31215 36020-36176
Q.plantF	Subset 9	4382-4836 8359-8967 9293-9672 13098-13328 16027-16240 19073-19185 38488-38980 38981-39326
LG	Subset 10	5525-7667
Q.yeast	Subset 11	8968-9292 13049-13097 15745-15791 30662-30807 39327-39821
mtZOAF	Subset 12	9673-9907 9908-10105 10378-10422 10423-11252 12481-12610 14387-14942 14943-15398 15645-15698 15699-15744 15792-15881 15979-16026
LGF	Subset 13	10164-10299 16921-17331 18597-18764 19849-20090 20091-20598 30962-31118 31216-31429 37223-38487
Q.yeast	Subset 14	11253-12480 16415-16920
LG	Subset 15	13362-13754
Q.yeast	Subset 16	13755-14386 15399-15489
Q.yeast	Subset 17	17861-18110 18765-18924 18925-19072 19186-19507 31895-32233 32234-33390 33391-35418 36606-37009
Q.yeast	Subset 18	19646-19744 19745-19796 21409-23811 25510-27081

Table S22. Partitions and substitution models proposed by Partition finder (Lanfear et al., 2017) for RAxML (Stamatakis, 2014) and ClipKIT gappy algorithm (Steenwyk et al., 2020).

Model	Partition no.	Partition range
LG	Subset1	1-505
LG	Subset2	506-981
CPREV	Subset3	982-1168
CPREV	Subset4	1169-1305 11315-11378
CPREV	Subset5	11869-12186 1306-1491
CPREV	Subset6	1492-1654
CPREV	Subset7	1655-1737 10728-10771
CPREV	Subset8	1738-1987 20366-20495 21095-21187
LG	Subset9	12187-12325 12448-12586 1988-2291
CPREV	Subset10	2292-2736 23484-23915
CPREV	Subset11	13450-13536 2737-3061
LG	Subset12	3062-3415
LG	Subset13	3416-4344 6554-6665
LG	Subset14	4345-4971
CPREV	Subset15	4972-5504
LG	Subset16	5505-5821 24179-24587
CPREV	Subset17	24588-24772 5822-6141
LG	Subset18	6142-6356
CPREV	Subset19	10999-11041 10772-10838 6357-6516
CPREV	Subset20	6517-6553 11074-11237
CPREV	Subset21	8669-8705 20931-21017 20622-20748 11278-11314 6666-6696 22311-22442 22155-22310
JTT	Subset22	13936-13983 6697-6738
LG	Subset23	6739-6768
LG	Subset24	6769-7520 9806-10292
LG	Subset25	11379-11868 7521-8254
LG	Subset26	8255-8335
CPREV	Subset27	11238-11277 8336-8477
LG	Subset28	8478-8668
MTREV	Subset29	8706-8748
CPREV	Subset30	22443-22580 20257-20365 8907-8936 8749-8906
LG	Subset31	8937-9296
LG	Subset32	9297-9805
CPREV	Subset33	10293-10643
LG	Subset34	10961-10998 10644-10727
CPREV	Subset35	10839-10876
LG	Subset36	11042-11073 10877-10915
LG	Subset37	10916-10960
LG	Subset38	12326-12447
CPREV	Subset39	13346-13449 21430-21653 13224-13345 12587-12712
CPREV	Subset40	12713-12988
CPREV	Subset41	12989-13107 20849-20930 21188-21429
LG	Subset42	13108-13223
LG	Subset43	13537-13745
CPREV	Subset44	21654-21859 13746-13821
CPREV	Subset45	13822-13887
CPREV	Subset46	14222-14402 13888-13935 22581-23483

Model	Partition no.	Partition range
LG	Subset47	13984-14221
CPREV	Subset48	14403-14725
CPREV	Subset49	14726-16219
LG	Subset50	16220-17917
CPREV	Subset51	17918-18669
CPREV	Subset52	18670-20256
CPREV	Subset53	20496-20621
LG	Subset54	20749-20848
CPREV	Subset55	21018-21094
LG	Subset56	21860-22046
LG	Subset57	22047-22154
CPREV	Subset58	24773-24962 23916-24178

Table S23. Partitions and substitution models proposed by Partition finder (Lanfear et al., 2017) for RAxML (Stamatakis, 2014) and ClipKIT kpic algorithm (Steenwyk et al., 2020).

Model	Partition no.	Partition range
LG	Subset1	1-452
LG	Subset2	453-814 22858-23211
CPREV	Subset3	815-1047
CPREV	Subset4	1048-1191 10308-10371 6335-6363
CPREV	Subset5	10818-11145 1192-1378
CPREV	Subset6	1379-1540
CPREV	Subset7	9800-9858 9767-9799 1541-1598
CPREV	Subset8	6206-6238 7725-7856 1599-1816
LG	Subset9	11372-11494 19751-19838 11146-11265 1817-2099
CPREV	Subset10	2100-2536
CPREV	Subset11	12824-12870 2537-2864
LG	Subset12	2865-3188
LG	Subset13	3189-4204
LG	Subset14	4205-4811
CPREV	Subset15	4812-5342
JTT	Subset16	5343-5615
CPREV	Subset17	5616-5911
LG	Subset18	5912-6094
LG	Subset19	6095-6205
LG	Subset20	6239-6334
JTT	Subset21	12871-12913 6364-6398
LG	Subset22	6399-6426
LG	Subset23	6427-7073 9059-9476
LG	Subset24	7074-7672
LG	Subset25	7673-7724 8652-9058 9971-9998
LG	Subset26	7857-8043 10076-10228
CPREV	Subset27	10229-10269 10270-10307 8044-8083 8299-8328 20816-20978
MTREV	Subset28	8084-8127
CPREV	Subset29	8128-8298 20319-20520 20979-21104 18940-19058
LG	Subset30	8329-8651
CPREV	Subset31	9477-9695
LG	Subset32	9696-9766
CPREV	Subset33	9859-9896
LG	Subset34	9897-9929
LG	Subset35	9930-9970 11266-11371
CPREV	Subset36	9999-10048
LG	Subset37	10049-10075
LG	Subset38	10372-10817
CPREV	Subset39	11495-11632 20073-20318
LG	Subset40	11633-11886 12668-12755 12469-12667
CPREV	Subset41	11887-11995
LG	Subset42	11996-12127
CPREV	Subset43	12263-12375 12128-12262 19490-19568 19839-20072
CPREV	Subset44	12756-12823 12376-12468
LG	Subset45	12914-13138
CPREV	Subset46	13139-13318 16484-17297
CPREV	Subset47	22140-22589 13319-13719

Model	Partition no.	Partition range
CPREV	Subset48	13720-15187
LG	Subset49	15188-16483
CPREV	Subset50	17298-18939
CPREV	Subset51	19059-19183 19569-19653
CPREV	Subset52	19184-19288
LG	Subset53	19289-19402 20521-20703
LG	Subset54	19403-19489
CPREV	Subset55	23392-23592 19654-19750 22590-22857
LG	Subset56	20704-20815
CPREV	Subset57	21105-21239
CPREV	Subset58	21240-22139
CPREV	Subset59	23212-23391

Table S24. Partitions and substitution models proposed by Partition finder (Lanfear et al., 2017) for RAxML (Stamatakis, 2014) and ClipKIT kpic-smart-gap algorithm (Steenwyk et al., 2020).

Model	Partition no.	Partition range
LG	Subset1	1-447
LG	Subset2	448-796
CPREV	Subset3	797-1002
CPREV	Subset4	1003-1146 6171-6199 10123-10186
CPREV	Subset5	1147-1332 10633-10946
CPREV	Subset6	1333-1494
CPREV	Subset7	9620-9678 1495-1550 9587-9619
CPREV	Subset8	6043-6075 7561-7689 1551-1768
LG	Subset9	11173-11295 19056-19141 1769-2043 10947-11066
CPREV	Subset10	2044-2477
CPREV	Subset11	12605-12650 2478-2786
LG	Subset12	2787-3105
LG	Subset13	3106-4061
LG	Subset14	4062-4659
CPREV	Subset15	4660-5180
JTT	Subset16	5181-5453
CPREV	Subset17	5454-5748
LG	Subset18	5749-5931
LG	Subset19	5932-6042
LG	Subset20	6076-6170
JTT	Subset21	6200-6234 12651-12692
LG	Subset22	6235-6262
LG	Subset23	8882-9299 6263-6909
LG	Subset24	6910-7508
LG	Subset25	7509-7560 8475-8881 9791-9817
LG	Subset26	7690-7876 9892-10043
CPREV	Subset27	10044-10084 10085-10122 7877-7913 8122-8151 20108-20270
MTREV	Subset28	7914-7957
CPREV	Subset29	19617-19814 7958-8121 18261-18376 20271-20395
LG	Subset30	8152-8474
CPREV	Subset31	9300-9515
LG	Subset32	9516-9586
CPREV	Subset33	9679-9716
LG	Subset34	9717-9749
LG	Subset35	11067-11172 9750-9790
CPREV	Subset36	9818-9867
LG	Subset37	9868-9891
LG	Subset38	10187-10632
CPREV	Subset39	19371-19616 11296-11433
LG	Subset40	12449-12536 11434-11687
CPREV	Subset41	11688-11795
LG	Subset42	11796-11927 12253-12448
CPREV	Subset43	11928-12051 18806-18884 19142-19370
CPREV	Subset44	12052-12161 13469-14566
CPREV	Subset45	12162-12252 12537-12604
LG	Subset46	12693-12915
CPREV	Subset47	15863-16618 12916-13095

Model	Partition no.	Partition range
CPREV	Subset48	13096-13468 21416-21857
LG	Subset49	14567-15862
CPREV	Subset50	16619-18260
CPREV	Subset51	18885-18969 18377-18501
CPREV	Subset52	18502-18604
LG	Subset53	19815-19997 18605-18718
LG	Subset54	18719-18805
CPREV	Subset55	22658-22848 18970-19055 21858-22123
LG	Subset56	19998-20107
CPREV	Subset57	20396-20526
CPREV	Subset58	20527-21415
LG	Subset59	22124-22477
CPREV	Subset60	22478-22657

Table S25. Partitions and substitution models proposed by Partition finder (Lanfear et al., 2017) for RAxML (Stamatakis, 2014) and ClipKIT smart-gap algorithm (Steenwyk et al., 2020).

Model	Partition no.	Partition range
LG	Subset1	1-530
LG	Subset2	531-1040
CPREV	Subset3	1041-1251
CPREV	Subset4	1252-1418 12449-12514
CPREV	Subset5	13012-13387 1419-1614
CPREV	Subset6	1615-1787
CPREV	Subset7	11831-11913 1788-1880 11785-11830
CPREV	Subset8	22286-22448 1881-2145
LG	Subset9	2146-2498
CPREV	Subset10	2499-2961
CPREV	Subset11	14815-14917 2962-3303
LG	Subset12	3304-3724
LG	Subset13	3725-5164
LG	Subset14	5165-5829
CPREV	Subset15	9618-9659 22891-22980 22578-22707 5830-6370 23809-24043
LG	Subset16	6371-6686 26662-27081
CPREV	Subset17	27082-27295 6687-7008
LG	Subset18	7009-7230
CPREV	Subset19	12082-12147 7231-7401
CPREV	Subset20	7402-7439 12189-12361
LG	Subset21	7440-7554 9409-9617
CPREV	Subset22	7555-7585 12405-12448 24393-24613 24614-24754
JTT	Subset23	15393-15440 7586-7627
LG	Subset24	7628-7659 12148-12188
LG	Subset25	10839-11342 7660-8414
LG	Subset26	12515-13011 8415-9172
LG	Subset27	11955-11994 9173-9258 12041-12081
CPREV	Subset28	12362-12404 9259-9408
MTREV	Subset29	9660-9705
CPREV	Subset30	24755-24896 22168-22285 9888-9919 9706-9887
LG	Subset31	9920-10295
LG	Subset32	10296-10838
CPREV	Subset33	11343-11695
LG	Subset34	11696-11784
CPREV	Subset35	11914-11954
LG	Subset36	11995-12040
LG	Subset37	13388-13528
LG	Subset38	13529-13650
LG	Subset39	13651-13796 23101-23200
CPREV	Subset40	23487-23808 14545-14686 13797-13966
CPREV	Subset41	13967-14260
CPREV	Subset42	15147-15254 14261-14389 22808-22890 23201-23486
LG	Subset43	14390-14544
CPREV	Subset44	14687-14814
LG	Subset45	14918-15146
CPREV	Subset46	15255-15343
CPREV	Subset47	24897-25896 15344-15392

Model	Partition no.	Partition range
LG	Subset48	15441-15676
CPREV	Subset49	15677-15894 19249-20109
CPREV	Subset50	15895-16347 25897-26359
CPREV	Subset51	16348-17560
LG	Subset52	17561-19248
CPREV	Subset53	20110-22167
CPREV	Subset54	22449-22577
LG	Subset55	22708-22807 24044-24280
CPREV	Subset56	22981-23100
LG	Subset57	24281-24392
CPREV	Subset58	27296-27506 26360-26661

Table S26. Partitions and substitution models proposed by Partition finder (Lanfear et al., 2017) for RAxML (Stamatakis, 2014) and trimAl automated1 algorithm (Capella-Gutiérrez et al., 2009).

Model	Partition no.	Partition range
LGF	Subset1	1-502 7764-7905
LG	Subset2	503-965
LGF	Subset3	966-1129 7906-8089 5284-5603
CPREV	Subset4	1130-1243
JTTF	Subset5	1385-1507 1244-1384
LGF	Subset6	1508-1588
LGF	Subset7	1589-1813 10600-10637 6122-6143
LG	Subset8	1814-2095
LGF	Subset9	2096-2454 13361-13509
CPREVF	Subset10	20464-20765 2455-2715
LGF	Subset11	2716-3067 18044-18164 21382-21541 18452-18540 18541-18759 4447-4968 13149-13360 12931-12999
LG	Subset12	3068-3878 11754-11887
LGF	Subset13	3879-4446
LGF	Subset14	10099-10140 4969-5283 11973-12246 11635-11753 13102-13148
LGF	Subset15	10206-10242 9665-10015 9195-9664 5604-5818
LGF	Subset16	10141-10205 6181-6209 5819-5977 10243-10281
CPREVF	Subset17	5978-6013 8090-8121 10638-10672 8122-8162 6144-6180 8305-8333 10673-10724 8163-8304
LGF	Subset18	21542-21708 6014-6121 10437-10599 20766-20973
MTARTF	Subset19	10282-10325 6210-6951
LG	Subset20	10725-11211 6952-7683
LGF	Subset21	10326-10363 7684-7763 8334-8690
LG	Subset22	8691-9194
LG	Subset23	10016-10098
LGF	Subset24	10364-10405 13765-14612 16275-16769
LG	Subset25	10406-10436
JTTF	Subset26	11212-11496 19265-19348
LGF	Subset27	19349-19503 17801-17920 11497-11634 19127-19264
LGF	Subset28	12359-12448 11888-11972 12647-12724
CPREV	Subset29	12247-12358
LGF	Subset30	18392-18451 19504-19633 12449-12562 12563-12646 18927-19126 12725-12930
LGF	Subset31	18165-18263 19746-20463 16770-17714 13000-13062
LGF	Subset32	13063-13101 18320-18391 13510-13764
LG	Subset33	14613-16274
CPREV	Subset34	17715-17800
CPREV	Subset35	17921-18043
LG	Subset36	18264-18319
CPREV	Subset37	18760-18926 19634-19745
LG	Subset38	20974-21381

Table S27. Partitions and substitution models proposed by Partition finder (Lanfear et al., 2017) for RAxML (Stamatakis, 2014) and trimAl gappyout algorithm (Capella-Gutiérrez et al., 2009).

Model	Partition no.	Partition range
LGF	Subset1	1-502 7968-8109
LG	Subset2	503-965
JTTF	Subset3	966-1149 20360-20454
CPREV	Subset4	1150-1275
JTTF	Subset5	1452-1591 1276-1451
LGF	Subset6	1592-1672
LGF	Subset7	6169-6204 1673-1897 10804-10842 10641-10803 8367-8508
LG	Subset8	1898-2179
CPREVF	Subset9	2180-2596 8294-8325
CPREVF	Subset10	8509-8537 22924-23102 2597-2906 21758-22110
LGF	Subset11	19036-19156 10530-10567 2907-3258 22764-22923 19157-19255 4638-5159 13430-13665
LG	Subset12	11977-12110 3259-4069
LGF	Subset13	4070-4637
LGF	Subset14	10303-10344 13339-13382 5160-5474 12220-12493 11858-11976 13383-13429
LGF	Subset15	6205-6312 8110-8293 5475-5794
LGF	Subset16	9869-10219 5795-6009 9399-9868
LGF	Subset17	10345-10409 10410-10446 6010-6168 10447-10485 10843-10877 8326-8366
JTTF	Subset18	6343-6384 6313-6342 10878-10935
MTART	Subset19	6385-6413
MTARTF	Subset20	10486-10529 6414-7155
LG	Subset21	10936-11422 7156-7887
LGF	Subset22	7888-7967 8538-8894
LG	Subset23	8895-9398
LG	Subset24	10220-10302
CPREV	Subset25	10568-10609
LG	Subset26	10610-10640
JTTF	Subset27	19333-19410 11423-11719
LGF	Subset28	18793-18912 11720-11857 20455-20609
LGF	Subset29	12923-13000 12606-12709 19411-19479 12111-12219 20610-20739 12710-12823 19788-19992 19993-20192 13001-13206
CPREV	Subset30	12494-12605
LGF	Subset31	12824-12922 20869-21757 13666-13843
LGF	Subset32	19256-19332 13207-13275 13844-14147
LGF	Subset33	19480-19568 13276-13338 14148-15205 19569-19787
LG	Subset34	15206-16867
CPREV	Subset35	16868-17493
LGF	Subset36	17494-18690
CPREV	Subset37	20740-20868 18691-18792
CPREV	Subset38	18913-19035
LGF	Subset39	22111-22355 20193-20359
LG	Subset40	22356-22763

Table S28. Partitions and substitution models proposed by Partition finder (Lanfear et al., 2017) for RAxML (Stamatakis, 2014) and trimAl strict algorithm (Capella-Gutiérrez et al., 2009).

Model	Partition no.	Partition range
LGF	Subset1	10308-10425 9315-9453 1-434
CPREV	Subset2	435-843
LGF	Subset3	844-1007 19721-19887
CPREV	Subset4	1008-1121
JTTF	Subset5	1263-1385 1122-1262
WAGF	Subset6	1386-1456
LGF	Subset7	1457-1649 7295-7419 9454-9491 5468-5489 7262-7294 9492-9521 9150-9186
LG	Subset8	1650-1889
LGF	Subset9	1890-2248 11986-12134
CPREVF	Subset10	7420-7445 2249-2509 18719-19020
CPREV	Subset11	2510-2793
LG	Subset12	2794-3471
LGF	Subset13	5382-5467 3472-3993 7072-7237 4760-5021
CPREV	Subset14	11158-11248 3994-4469
LGF	Subset15	11736-11773 4470-4759 11697-11735 10734-10973 10974-11067
MTARTF	Subset16	9286-9314 9028-9060 9187-9223 8221-8648 5550-6240 5022-5214
CPREV	Subset17	5215-5350
MTART	Subset18	5351-5381
JTTF	Subset19	9118-9149 9522-9573 5490-5526
MTART	Subset20	5527-5549
LG	Subset21	6241-6889
LG	Subset22	6890-6963
LG	Subset23	6964-7071 13238-14704
MTARTF	Subset24	7238-7261 8950-9027
LGF	Subset25	7446-7758
LGF	Subset26	9224-9251 8649-8949 7759-8220
CPREV	Subset27	9061-9117
LGF	Subset28	10530-10648 16325-16432 10426-10529 9252-9285
LG	Subset29	9574-10022
JTTF	Subset30	10023-10307 17560-17643
LGF	Subset31	16145-16230 17087-17253 17254-17421 11249-11332 17772-17888 10649-10733 17644-17771 16682-16753 11774-11985
LGF	Subset32	11068-11157 11578-11645 12135-12389
LGF	Subset33	11333-11401 16754-16813 16539-16625 11646-11696 15200-16144 18001-18718
LGF	Subset34	16814-16888 17422-17559 12390-13237 16889-17086 11402-11577
CPREV	Subset35	14705-15199
MTARTF	Subset36	19021-19228 16231-16324
LG	Subset37	16433-16538
LG	Subset38	16626-16681
CPREV	Subset39	17889-18000
LG	Subset40	19229-19582
CPREV	Subset41	19583-19720

Table S29. Partitions and substitution models proposed by Partition finder (Lanfear et al., 2017) for RAxML (Stamatakis, 2014) and original alignment (no trimming strategy).

Model	Partition no.	Partition range
LGF	Subset1	12611-12775 1-611
LG	Subset2	612-1534
LGF	Subset3	1535-2057 12776-13003 9293-9672
CPREVF	Subset4	2058-2242 40061-40342
JTTF	Subset5	2461-2647 2243-2460
LGF	Subset6	14943-15398 2648-2837
LGF	Subset7	16241-16292 2838-3143 13004-13048 10164-10299 13098-13328 16027-16240
LG	Subset8	17699-17860 3144-3873
CPREVF	Subset9	18765-18924 3874-4381
CPREVF	Subset10	38488-38980 13329-13361 38981-39326 4382-4836
LG	Subset11	4837-5524
LG	Subset12	5525-7667
LGF	Subset13	7668-8358
LGF	Subset14	17332-17482 8359-8967 30962-31118 39822-40060 19745-19796 19849-20090 35419-36019 33391-35418 19186-19507 36606-37009 31728-31894
JTTF	Subset15	30662-30807 8968-9292 15645-15698 30093-30661
LGF	Subset16	15399-15489 14387-14942 9673-9907
LGF	Subset17	10378-10422 15699-15744 9908-10105 16293-16337 13049-13097
MTART	Subset18	10106-10163
JTTF	Subset19	10332-10377 10300-10331 16338-16414
MTARTF	Subset20	15745-15791 10423-11252
LG	Subset21	16415-16920 11253-12480
LGF	Subset22	13362-13754 12481-12610
LG	Subset23	13755-14386
CPREV	Subset24	15490-15539 15540-15644
LGF	Subset25	15792-15881 19797-19848 17483-17698 18111-18444
CPREVF	Subset26	15882-15978 25510-27081
LG	Subset27	15979-16026
JTTF	Subset28	36020-36176 16921-17331 29961-30092 18925-19072 31216-31429 31430-31727
LGF	Subset29	31119-31215 17861-18110 31895-32233
MTREVF	Subset30	18445-18596 37010-37222 32234-33390
LGF	Subset31	19073-19185 18597-18764
LGF	Subset32	20599-21408 19508-19645
JTTF	Subset33	21409-23811 19646-19744
LGF	Subset34	37223-38487 20091-20598
LG	Subset35	23812-25509
JTTF	Subset36	27082-29960
CPREV	Subset37	30808-30961
LGF	Subset38	36177-36605
LG	Subset39	39327-39821

References

Bengtson, S., Sallstedt, T., Belivanova, V., & Whitehouse, M. (2017). Three-dimensional preservation of cellular and subcellular structures suggests 1.6 billion-year-old crown-group red algae. *PLoS Biology*, *15*(3), e2000735.

Bodył, A. (2018). Did some red alga-derived plastids evolve via kleptoplastidy? A hypothesis. *Biological Reviews*, *93*(1), 201–222. https://doi.org/10.1111/brv.12340

Bodył, A., Stiller, J. W., & Mackiewicz, P. (2009). Chromalveolate plastids: Direct descent or multiple endosymbioses? *Trends in Ecology & Evolution*, *24*(3), 119–121. https://doi.org/10.1016/j.tree.2008.11.003

Bouckaert, R., Heled, J., Kühnert, D., Vaughan, T., Wu, C.-H., Xie, D., Suchard, M. A., Rambaut, A., & Drummond, A. J. (2014). BEAST 2: A Software Platform for Bayesian Evolutionary Analysis. *PLOS Computational Biology*, *10*(4), e1003537. https://doi.org/10.1371/journal.pcbi.1003537

Bown, P. R., Lees, J. A., & Young, J. R. (2004). Calcareous nannoplankton evolution and diversity through time. In H. R. Thierstein & J. R. Young (Eds.), *Coccolithophores: From Molecular Processes to Global Impact* (pp. 481–508). Springer. https://doi.org/10.1007/978-3-662-06278-4_18

Capella-Gutiérrez, S., Silla-Martínez, J. M., & Gabaldón, T. (2009). trimAl: A tool for automated alignment trimming in large-scale phylogenetic analyses. *Bioinformatics*, *25*(15), Article 15. https://doi.org/10.1093/bioinformatics/btp348

Gagat, P., Bodył, A., Mackiewicz, P., & Stiller, J. W. (2014). Tertiary Plastid Endosymbioses in Dinoflagellates. In W. Löffelhardt (Ed.), *Endosymbiosis* (pp. 233–290). Springer. https://doi.org/10.1007/978-3-7091-1303-5_13

Gibson, T. M., Shih, P. M., Cumming, V. M., Fischer, W. W., Crockford, P. W., Hodgskiss, M. S., Wörndle, S., Creaser, R. A., Rainbird, R. H., & Skulski, T. M. (2018). Precise age of Bangiomorpha pubescens dates the origin of eukaryotic photosynthesis. *Geology*, *46*(2), 135–138.

Hadariová, L., Vesteg, M., Hampl, V., & Krajčovič, J. (2018). Reductive evolution of chloroplasts in non-photosynthetic plants, algae and protists. *Current Genetics*, *64*(2), 365–387. https://doi.org/10.1007/s00294-017-0761-0

Kaczmarska, I., Jr, B. S. G., Ehrman, J. M., & Thaler, M. (2017). Sexual reproduction in plagiogrammacean diatoms: First insights into the early pennates. *PLOS ONE*, *12*(8), e0181413. https://doi.org/10.1371/journal.pone.0181413

Kalyaanamoorthy, S., Minh, B. Q., Wong, T. K. F., von Haeseler, A., & Jermiin, L. S. (2017). ModelFinder: Fast model selection for accurate phylogenetic estimates. *Nature Methods*, *14*(6), Article 6. https://doi.org/10.1038/nmeth.4285

Kooistra, W. H., & Medlin, L. K. (1996). Evolution of the diatoms (Bacillariophyta): IV. A reconstruction of their age from small subunit rRNA coding regions and the fossil record. *Molecular Phylogenetics and Evolution*, *6*(3), 391–407.

Lanfear, R., Frandsen, P. B., Wright, A. M., Senfeld, T., & Calcott, B. (2017). PartitionFinder 2: New Methods for Selecting Partitioned Models of Evolution for Molecular and Morphological Phylogenetic Analyses. *Molecular Biology and Evolution*, *34*(3), 772–773. https://doi.org/10.1093/molbev/msw260

Mansour, J. S., & Anestis, K. (2021). Eco-Evolutionary Perspectives on Mixoplankton. *Frontiers in Marine Science*, *8*. https://www.frontiersin.org/articles/10.3389/fmars.2021.666160

Medlin, L. K. (2015). A timescale for diatom evolution based on four molecular markers: Reassessment of ghost lineages and major steps defining diatom evolution. *Vie et Milieu-Life and enVironment*.

Minh, B. Q., Schmidt, H. A., Chernomor, O., Schrempf, D., Woodhams, M. D., von Haeseler, A., & Lanfear, R. (2020). IQ-TREE 2: New Models and Efficient Methods for Phylogenetic Inference in the Genomic Era. *Molecular Biology and Evolution*, *37*(5), 1530–1534. https://doi.org/10.1093/molbev/msaa015

Pietluch, F., Mackiewicz, P., Sidorczuk, K., & Gagat, P. (2022). Dating the photosynthetic organelle evolution in Archaeplastida, Paulinella and secondary-plastid bearing lineages. *bioRxiv*, 2022–07.

Ronquist, F., Teslenko, M., van der Mark, P., Ayres, D. L., Darling, A., Höhna, S., Larget, B., Liu, L., Suchard, M. A., & Huelsenbeck, J. P. (2012). MrBayes 3.2: Efficient Bayesian Phylogenetic Inference and Model Choice Across a Large Model Space. *Systematic Biology*, *61*(3), 539–542. https://doi.org/10.1093/sysbio/sys029

Sibbald, S. J., & Archibald, J. M. (2020). Genomic Insights into Plastid Evolution. *Genome Biology and Evolution*, *12*(7), 978–990. https://doi.org/10.1093/gbe/evaa096

Stamatakis, A. (2014). RAxML version 8: A tool for phylogenetic analysis and post-analysis of large phylogenies. *Bioinformatics*, *30*(9), 1312–1313. https://doi.org/10.1093/bioinformatics/btu033

Steenwyk, J. L., Iii, T. J. B., Li, Y., Shen, X.-X., & Rokas, A. (2020). ClipKIT: A multiple sequence alignment trimming software for accurate phylogenomic inference. *PLOS Biology*, *18*(12), e3001007. https://doi.org/10.1371/journal.pbio.3001007

Stiller, J. W., Schreiber, J., Yue, J., Guo, H., Ding, Q., & Huang, J. (2014). The evolution of photosynthesis in chromist algae through serial endosymbioses. *Nature Communications*, *5*(1), Article 1. https://doi.org/10.1038/ncomms6764

Strassert, J. F. H., Irisarri, I., Williams, T. A., & Burki, F. (2021). A molecular timescale for eukaryote evolution with implications for the origin of red algal-derived plastids. *Nature Communications*, *12*(1), Article 1. https://doi.org/10.1038/s41467-021-22044-z

Xiao, S., Knoll, A. H., Yuan, X., & Pueschel, C. M. (2004). Phosphatized multicellular algae in the Neoproterozoic Doushantuo Formation, China, and the early evolution of florideophyte red algae. *American Journal of Botany*, *91*(2), 214–227.
